# Structure and Conductivity in LISICON Analogues within the Li_4_GeO_4_–Li_2_MoO_4_ System

**DOI:** 10.1021/acs.inorgchem.3c01222

**Published:** 2023-07-14

**Authors:** Ludan Zhang, Marcin Malys, Jan Jamroz, Franciszek Krok, Wojciech Wrobel, Stephen Hull, Haixue Yan, Isaac Abrahams

**Affiliations:** † Department of Chemistry, Queen Mary University of London, Mile End Road, London E1 4NS, U.K.; ‡ Shenzhen CAPCHEM Technology Company Limited, Pingshan District, Shenzhen 518118, China; § Faculty of Physics, Warsaw University of Technology, Koszykowa 75, Warszawa 00-662, Poland; ∥ Science and Technology Facilities Council, ISIS Facility, Rutherford Appleton Laboratory, Chilton, Didcot, Oxofordshire OX11 OQX, U.K.; ⊥ School of Engineering and Materials Science, Queen Mary University of London, Mile End Road, London E1 4NS, U.K.

## Abstract

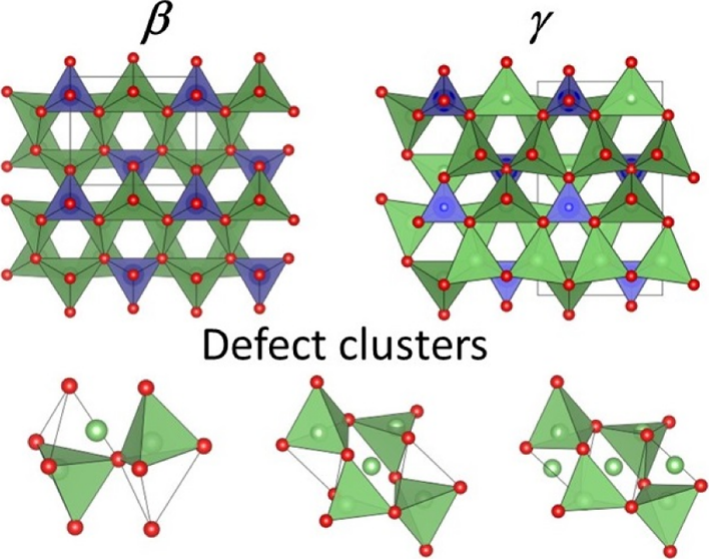

New solid electrolytes are crucial for the development of all-solid-state lithium batteries with advantages in safety and energy densities over current liquid electrolyte systems. While some of the best solid-state Li^+^-ion conductors are based on sulfides, their air sensitivity makes them less commercially attractive, and attention is refocusing on air-stable oxide-based systems. Among these, the LISICON-structured systems, such as Li_2+2*x*_Zn_1–*x*_GeO_4_ and Li_3+*x*_V_1–*x*_Ge_*x*_O_4_, have been relatively well studied. However, other systems such as the Li_4_GeO_4_–Li_2_MoO_4_ system, which also show LISICON-type structures, have been relatively little explored. In this work, the Li_4–2*x*_Ge_1–*x*_Mo_*x*_O_4_ solid solution is investigated systematically, including the solid solution limit, structural stability, local structure, and the corresponding electrical behavior. It is found that a γ-LISICON structured solution is formed in the range of 0.1 ≤ *x* < 0.4, differing in structure from the two end members, Li_4_GeO_4_ and Li_2_MoO_4_. With increasing Mo content, the β-phase becomes increasingly more stable than the γ-phase, and at *x* = 0.5, a pure β-phase (β-Li_3_Ge_0.5_Mo_0.5_O_4_) is readily isolated. The structure of this previously unknown compound is presented, along with details of the defect structure of Li_3.6_Ge_0.8_Mo_0.2_O_4_ (*x* = 0.2) based on neutron diffraction data. Two basic types of defects are identified in Li_3.6_Ge_0.8_Mo_0.2_O_4_ involving interstitial Li^+^-ions in octahedral sites, with evidence for these coming together to form larger defect clusters. The *x* = 0.2 composition shows the highest conductivity of the series, with values of 1.11 × 10^–7^ S cm^–1^ at room temperature rising to 5.02 × 10^–3^ S cm^–1^ at 250 °C.

## Abstract 2

A γ-LISICON-structured solution is formed in the Li_4−2*x*_Ge_1−*x*_Mo_*x*_O_4_ system in the range of 0.1 ≤ *x* < 0.4. Two basic types of defects are identified in the γ-phase involving interstitial Li^+^-ions in octahedral sites, with evidence for these coming together to form larger defect clusters. With increasing Mo content, the β-phase becomes increasingly more stable than the γ-phase, and at *x* = 0.5, a previously unknown β-phase (β-Li_3_Ge_0.5_Mo_0.5_O_4_) is readily isolated.

## 1 Introduction

The development of fast ion conducting solid electrolytes is crucial for the realization of all-solid-state lithium (ion) batteries, which are required to address two application issues in electric vehicle technology, namely, safety and mileage extension. Currently, among the main families of inorganic solid electrolytes, such as garnets,^1,2^ NASICONs,^3−6^ and thio-LISICONs,^7,8^ the thio-LISICONs stand out due to their superior lithium ionic conductivity at ambient temperature. A record-high ionic conductivity for a lithium-ion conducting solid of 0.012 S cm^–1^ at room temperature has been observed in Li_10_GeP_2_S_12_, which is even higher than that of commercial electrolytic liquids.^9^ However, commercialization of sulfide-based systems is hampered by the high costs of raw materials and the need for expensive inert gas processing conditions due to their air sensitivity. Thus, the development of low-cost oxide-based solid electrolytes with superior ionic conductivity is strongly desired.

The oxide-based LISICONs also show relatively high Li^+^-ion conductivity, especially at high temperatures, but at room temperature, the conductivity values are significantly lower than sulfide-based LISICONs, as well as other oxide systems based on the NASICON, perovskite, and garnet structures. Despite this, there is much to be gained from studies of the oxide-based LISICONs since they share many structural attributes with their highly conducting sulfide counterparts. More generally, the characterization of defects and defect clustering, previously identified in other LISICON systems, such as Li_2+2*x*_Zn_1–*x*_GeO_4_^10^ and Li_3+*x*_Ge_*x*_V_1–*x*_O_4_,^11^ can help in gaining a more fundamental understanding of ion migration in such complex oxide systems. The LISICON structure can be mainly thought of as being derived from that of γ-Li_3_PO_4_, with Li and P occupying half the tetrahedral sites in the distorted hexagonal close-packed structure. Subvalent cation substitution of P in Li_3_PO_4_ requires charge balance through the introduction of additional Li^+^-ions, which are located in the interstitial octahedral sites. Alternatively, in some cases, supervalent substitution of M cations in oxides of the type Li_4_MO_4_ (M = Si, Ge, Ti) can also lead to LISICON-structured solids of the type Li_4–*y*_M_*x*_N_1–*x*_O_4_, where three of the Li^+^-ions form part of the tetrahedral framework and the remainder, 1 – *y*, is the number of interstitial octahedral Li^+^-ions per formula unit, which is determined by the average charge of the M and N cations, such that *y* = [*x*·*n*_1_ + (1 – *x*)·*n*_2_] – 4, where *n*_1_ and *n*_2_ are the charges of  and  cations, respectively. For example, substitution of Ge^4+^ in Li_4_GeO_4_ by W^6+^ has been reported to yield a LISICON-type solid solution with a conductivity value of *ca.* 5 × 10^–5^ S cm^–1^ at room temperature in Li_3.7_Ge_0.85_W_0.15_O_4_.^12^ Despite the chemical similarities of tungsten and molybdenum, similar substitutions of Si^4+^ and Ge^4+^ by Mo^6+^ in Li_4_SiO_4_ and Li_4_GeO_4_, respectively, has been reported to yield compositions with the γ-LISICON structure but with lower room temperature conductivities of around 10^–7^ S cm^–1^.^13,14^ Little information is available on the Li_4–2*x*_Ge_1–*x*_Mo_*x*_O_4_ system, apart from a single composition *x* = 0.3.^14^

Based on these previous studies, it can be concluded that Mo^6+^ is likely to form a LISICON-type solid solution, in a similar way to W^6+^, through a classical solid-state reaction.^15^ If so, then questions arise as to how extensive this solid solution is, and how the structure and conductivity vary with composition compared to other LISICON systems. With these questions in mind, here, the Li_2_MoO_4_–Li_4_GeO_4_ system is systematically investigated. Electrical behavior is characterized using AC impedance spectroscopy, while a detailed structural analysis is presented using a combination of neutron and X-ray powder diffraction. A LISICON-structured solid solution of general formula Li_4–2*x*_Ge_1–*x*_Mo_*x*_O_4_ is found to form in the range of 0.1 ≤ *x* < 0.4, with the poorly conducting β-phase becoming increasingly more stable than the more highly conducting γ-phase with increasing *x*-value.

## 2 Experimental Section

### 2.1 Synthesis

Samples of composition Li_4–2*x*_Ge_1–*x*_Mo_*x*_O_4_ (0.0 ≤ *x* ≤ 1.0) were prepared using a classical solid-state reaction. Stoichiometric amounts of Li_2_CO_3_ (99%, BDH Chemicals), GeO_2_ (99.999%, Aldrich Gold), and MoO_3_ (99.5%, Sigma-Aldrich) were ground thoroughly in an agate mortar to form a homogeneous paste with methylated spirits. After drying the paste at 80 °C, the mixtures were heated in an alumina crucible at 650 °C for 1 h, followed by calcining at temperatures between 650 and 850 °C for various times up to 24 h. Samples were slowly cooled in the furnace to room temperature. Various conditions were investigated to optimize the purity of the different compositions, with specific details of those used to prepare the final compositions summarized in Table S1.

The as-prepared powders were used to make pellets through spark plasma sintering (SPS) (HPD 25/1, FCT, Rauenstein, Germany). Samples were pressed into a graphite die with a 10 mm diameter surrounded by carbon foil. The resulting powder was then sintered at 600 to 800 °C for 5 min at a uniaxial pressure of 60 MPa under vacuum. SPS-processed samples were subsequently annealed at 600 to 850 °C for 11–20 h to remove residual carbon arising from the carbon foil. Table S2 summarizes the sintering conditions used for each composition.

### 2.2 Characterization

X-ray powder diffraction (XRD) was used to characterize the phase purity and crystal structure of samples. XRD data were collected on a PANAlytical X’Pert Pro diffractometer, equipped with an X’Celerator detector, in θ/θ geometry using Ni-filtered Cu Kα radiation (λ = 1.5418 Å), over the 2θ range from 5 to 120° in steps of 0.0334°, with an effective count time of 200 s per step. Calibration was carried out with an external LaB_6_ standard. Elevated temperature measurements were performed using an Anton-Paar HTK 1200 high-temperature camera, with samples placed on a Pt-coated sample holder. Diffraction patterns were acquired at room temperature and at 50 °C intervals from 50 to 750 °C, over the 2θ range 5–120° in steps of 0.033°, with an effective scan time of 50 s per step. The microstructure of the ceramic pellets was examined using an FEI Inspect F scanning electron microscope.

Neutron diffraction data were collected at room temperature on the Polaris diffractometer at the ISIS Facility, Rutherford Appleton Laboratory. Data from the back-scattering (average angle 146.72°) and 90° (average angle 92.59°) banks were used in subsequent refinements. For room temperature measurements, the *x* = 0.0 and *x* = 1.0 samples were loaded into an 11 mm diameter thin-walled vanadium can, with data collections corresponding to 1000 μA h of proton beam charge. The *x* = 0.2 and 0.5 samples were sealed in a silica tube and placed inside a thin-walled vanadium can, with data collections corresponding to 1000 and 500 μA h of proton beam charge, respectively. For elevated temperature measurements on the *x* = 0.2 composition, data were collected at 50 °C intervals from 300 to 700 °C, with short data collections of 30 μA h carried out at the intermediate temperatures and a long data collection of 1000 μA h at 700 °C. Structural analysis based on the X-ray and neutron diffraction data was carried out using the Rietveld method with the GSAS suite of programs.^16^ The starting models were based on the structures of Li_3.5_Zn_0.5_GeO_4_,^17^ β-Li_3_PO_4_,^18^ and Li_2_MoO_4_.^19^

The density of ceramic pellets was measured based on the classical Archimedes method by displacement of water at room temperature. For impedance measurements, annealed ceramic pellets were first cut and polished into blocks of *ca.* 4 mm × 4 mm × 2 mm. Gold electrodes were sputtered by cathodic discharge. AC impedance spectroscopy was performed on a fully automated system, based on a Solartron 1255 frequency response analyzer, in conjunction with a bespoke automatic current/voltage converter. Impedance data were collected over the frequency range from 0.1 to 1 × 10^6^ Hz, in the approximate temperature range from 50 to 300 °C, over two cycles of heating and cooling. Fitting of impedance data was carried out using the program WFIRDARMM.^20,21^

## 3 Results and Discussion

### 3.1 Solid Solution Formation

**Figure 1:**
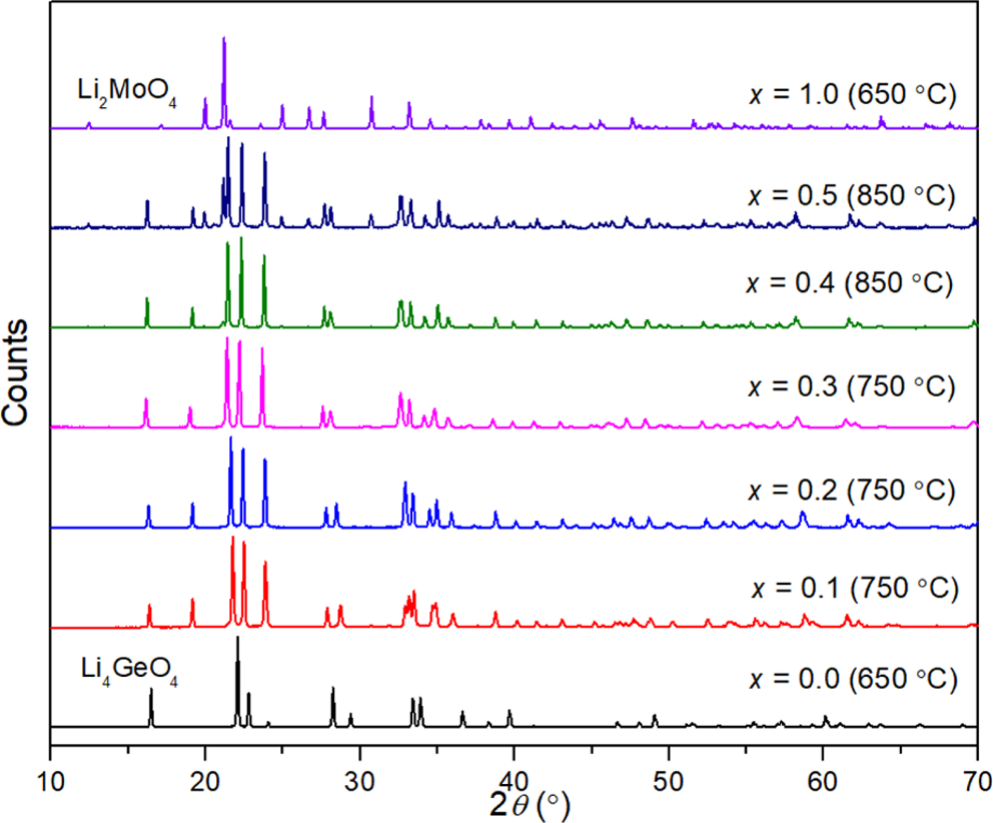
XRD patterns of studied compositions in the Li_4–2*x*_Ge_1–*x*_Mo_*x*_O_4_ system, with calcination temperatures given in brackets.

To explore solid solution formation in the Li_2_MoO_4_–Li_4_GeO_4_ system, the solid solution limit was first investigated. Since the study was carried out over a wide compositional range, optimum calcination temperatures were explored to ensure the purity of products at each composition. Unsurprisingly, over such a wide range of compositions, the optimum sintering temperature varied as summarized in Table S2. Figure 1 shows the XRD patterns for the 0.1 ≤ *x* ≤ 0.5 compositions and the two end members Li_2_MoO_4_ and Li_4_GeO_4_. As can be seen, all compositions exhibited good crystallinity. The intermediate compositions 0.1 ≤ *x* ≤ 0.5 exhibit XRD patterns consistent with the γ-LISICON structure and different from the two end members. At *x* = 0.4 and 0.5, peaks corresponding to the end member Li_2_MoO_4_ are evident.

**Figure 2:**
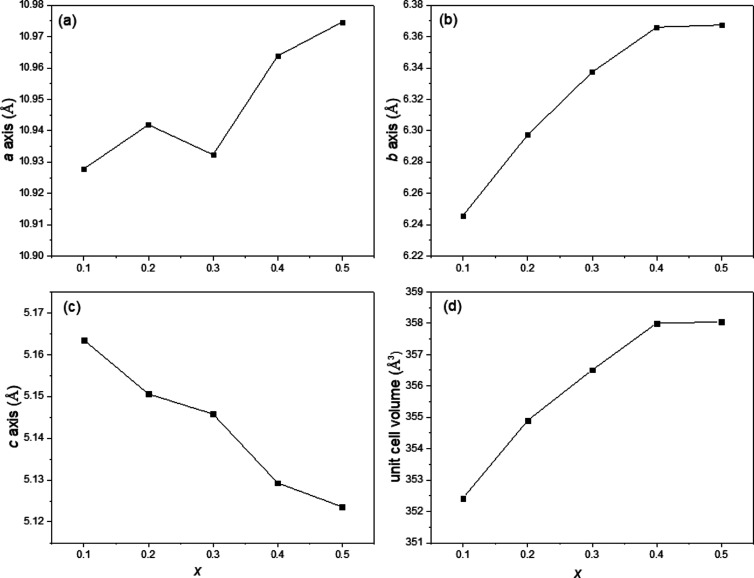
Compositional variation of unit cell parameters (a) *a*, (b) *b*, (c) *c*, and (d) unit cell volume in the Li_4–2*x*_Ge_1–*x*_Mo_*x*_O_4_ system. Error bars are smaller than the symbols used.

The refined unit cell parameters for the Li_4–2*x*_Ge_1–*x*_Mo_*x*_O_4_ system are listed in Table S3, with the corresponding plots in Figure 2. The unit cell volume in the Li_4–2*x*_Ge_1–*x*_Mo_*x*_O_4_ series shows a general increasing trend with increasing *x*-value over the range 0.1 ≤ *x* ≤ 0.4. This can be mainly attributed to the ionic radius of Mo^6+^ (0.41 Å), which is slightly larger than that of Ge^4+^ (0.39 Å) when both are in 4-coordinate geometry.^22^ This general trend is reflected in the compositional variation of the *a*- and *b*-axes, although with a notable local minimum in the *a*-axis parameter at *x* = 0.30. In contrast, the *c*-axis shows a gradual decrease with increasing *x*-value over the same compositional range. In the Li_4–2*x*_Ge_1–*x*_Mo_*x*_O_4_ system, to maintain charge balance with increased Mo^6+^ content in the (Li_3_Ge_1–*x*_Mo_*x*_O_4_)^(1–2*x*)–^ skeleton, vacancies are created on the interstitial octahedral lithium sites. At *x* = 0.5, the formula is Li_3_Ge_0.5_Mo_0.5_O_4_, and theoretically, no interstitial lithium ions are present. According to previous work,^17^ the interstitial octahedral lithium ions can cause the tetrahedral Li^+^-ions to be displaced to some extent along the *c*-axis. In other words, the less occupied the interstitial octahedral sites are, the less likely is the displacement of Li^+^-ions within the tetrahedral sites. Therefore, the gradual decrease in the *c*-axis with increasing *x*-value can be attributed to the decrease in displacement along the *c*-axis. The results suggest the gradual elimination of lithium interstitials with a solid solution limit for the γ-phase between *x* = 0.3 and 0.4.

**Figure 3:**
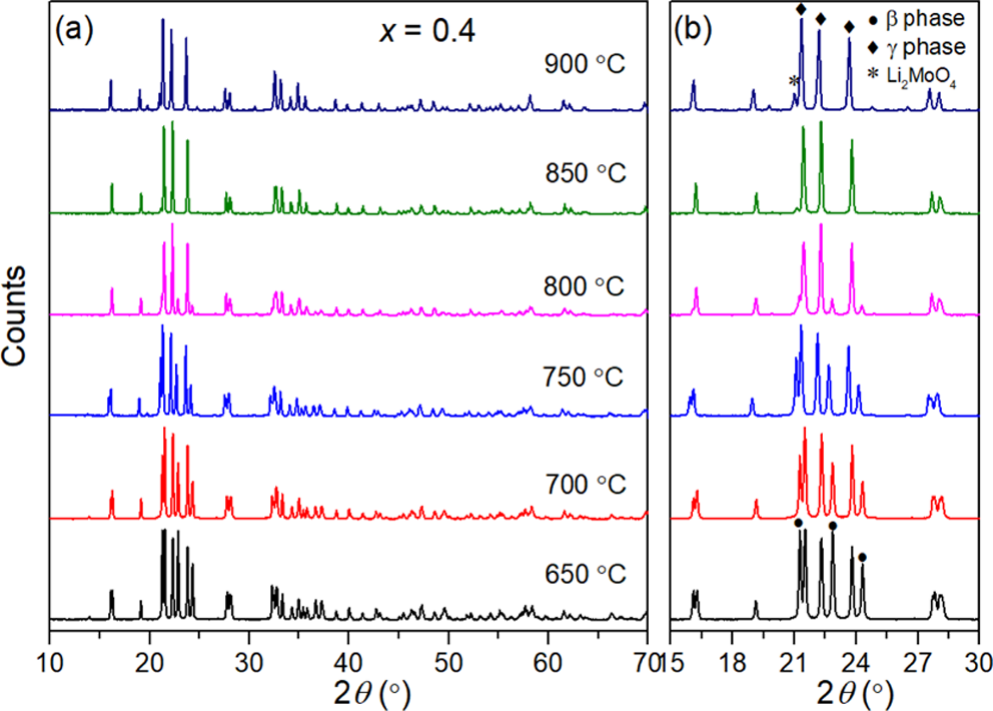
(a) XRD patterns of the *x* = 0.4 composition in the Li_4–2*x*_Ge_1–*x*_Mo_*x*_O_4_ system calcined at selected temperatures for 24 h, with detail shown in (b).

During the synthesis, it was noted that the β-LISICON structure in the space group *Pmn*2_1_ appeared at different stages for these compositions. At a calcination temperature of 750 °C, the *x* = 0.1, 0.2, and 0.3 compositions all yielded a pure γ-phase after 24 h. However, for the *x* = 0.4 composition, a mixture of β- and γ-phases was observed under the same conditions. Experiments on this composition at different calcination temperatures (Figure 3) showed that at 650 °C, the β-phase is relatively dominant, with the γ/β phase ratio increasing with increasing calcination temperature until at 850 °C, a complete transformation to the γ-phase is achieved, although accompanied by the appearance of a small amount of Li_2_MoO_4_. On increasing the calcination temperature further to 900 °C, increased formation of Li_2_MoO_4_ is seen. Therefore, a calcination temperature of 850 °C was used in the final preparation.

A similar study was carried out for the *x* = 0.5 composition (Figure S1). A pure β-phase is seen at 650 °C and remains the dominant phase on increasing temperature up to 800 °C. The amount of γ-phase increases over this temperature range, and on increasing the temperature to 850 °C, the γ-phase completely replaces the β-phase but is accompanied by the appearance of a significant amount of Li_2_MoO_4_. The powders were found to melt at temperatures above 850 °C. Comparing the temperature range for the existence of the β-phase and the γ-phase for compositions of 0.1 ≤ *x* ≤ 0.5, it can be concluded that, with increasing *x*-value in Li_4–2*x*_Ge_1–*x*_Mo_*x*_O_4_, the ratio of the β-phase to the γ-phase decreases with increasing temperature. To obtain the γ-phase in high *x*-value compositions, high calcination temperatures are required, but this comes at the expense of purity, with the appearance of Li_2_MoO_4_.

To see whether the β-phase solid solution extends beyond *x* = 0.5, the *x* = 0.6 composition was synthesized. The XRD pattern of this composition is shown in Figure S2. The sample shows a mixture of Li_2_MoO_4_ and a β-phase, presumed to be β-Li_3_Ge_0.5_Mo_0.5_O_4_. This is perhaps unsurprising, as in order to maintain a solid solution, vacancies would need to be introduced into the tetrahedral framework, which is likely to be energetically unfavorable.

### 3.2 Thermal Variation of Structure

**Figure 4:**
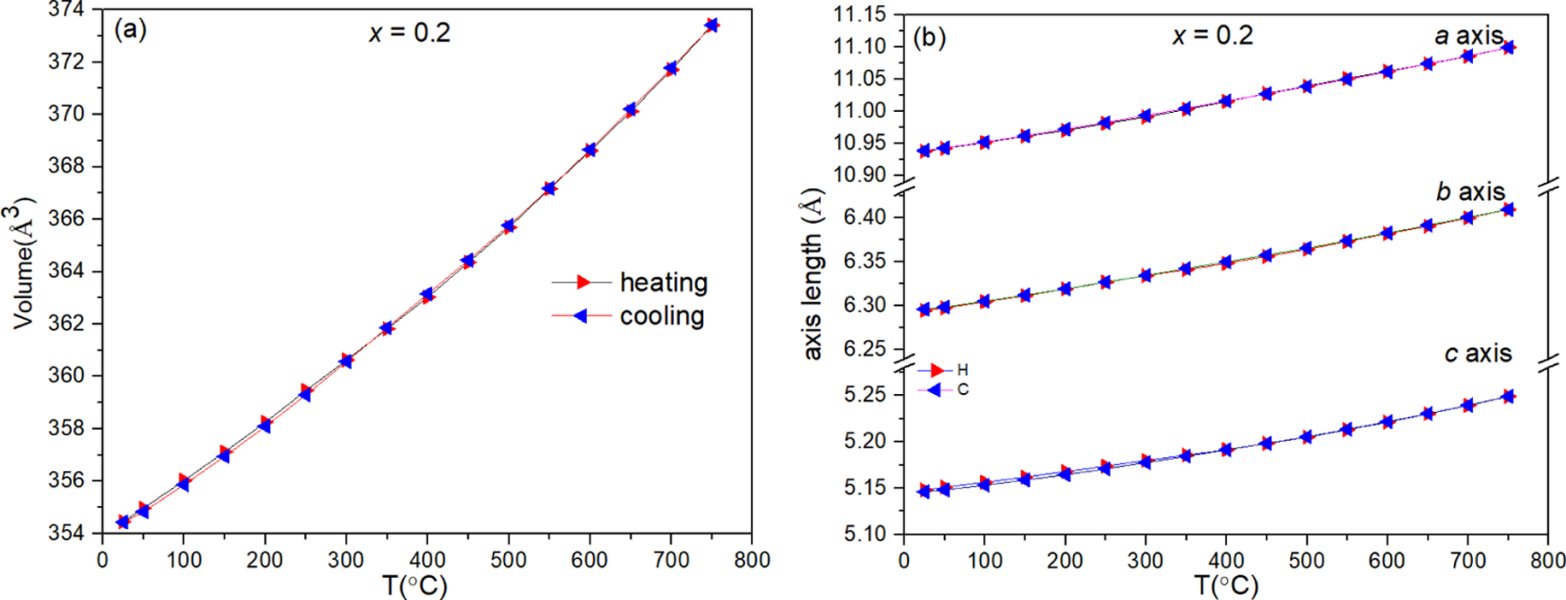
Thermal variation of (a) unit cell volume and (b) lattice parameters for Li_3.6_Ge_0.8_Mo_0.2_O_4_ (*x* = 0.2) on heating and cooling.

The diffraction patterns for compositions *x* = 0.1, 0.2, 0.3, 0.4, and 0.5 at selected temperatures up to 750 °C are given in Figures S3–S7. The thermal variation of unit cell volume and lattice parameters for the *x* = 0.2 composition is given in Figure 4 as a representative example, with those for other compositions summarized in Figure S8.

For the *x* = 0.1 composition, the diffraction patterns (Figure S3) show that the LISICON phase is preserved throughout heating and cooling. However, on heating up to 350 °C, a small amount of a second phase, Li_6_Ge_2_O_7_, appears. The amount of this second phase diminishes at 750 °C, and on cooling, the amount of Li_6_Ge_2_O_7_ remains at a negligible level down to room temperature. This phase separation is evident in the lattice parameter variation on heating (Figure S8), with the data showing a difference between heating and cooling curves below 400 °C. The *x* = 0.2 and 0.3 compositions (Figures S4 and S5) show no such phase separation in their diffraction patterns, although, for the *x* = 0.3 composition, there is a small difference in the lattice parameters below 400 °C on heating and cooling. For the *x* = 0.4 composition (Figure S6), although the γ-LISICON phase is seen throughout the heating and cooling regimes, on heating to 600 °C, the β-phase appears, and the amount of this secondary phase increases up to 750 °C. On cooling, the secondary β-phase is maintained at room temperature. For the *x* = 0.5 composition (Figure S7), the experiment was carried out beginning with a pure β-phase. This phase is preserved throughout heating and cooling, with no evidence of a transition to the γ-phase. The unit cell volume plots of all compositions are similar, showing two linear regions with a change in slope at around 450 to 500 °C.

**Figure 5:**
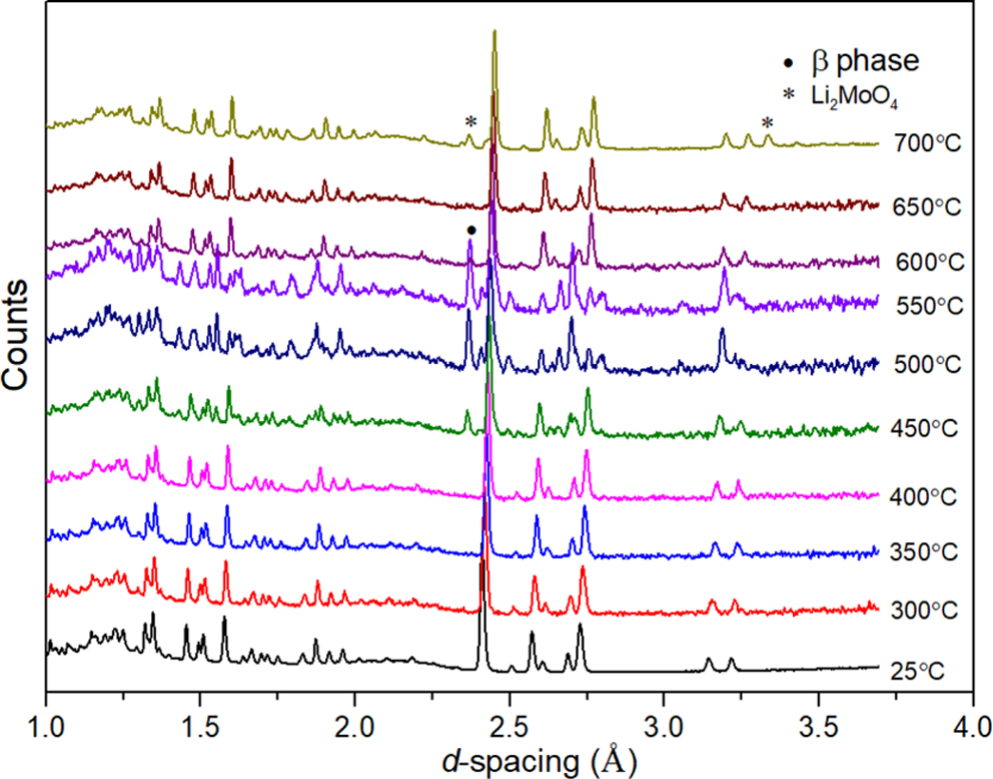
Neutron diffraction patterns (90° detector bank) for Li_3.6_Ge_0.8_Mo_0.2_O_4_ (*x* = 0.2) on heating.

To further examine the subtle changes in this system, a variable-temperature neutron diffraction experiment was carried out on the *x* = 0.2 composition from room temperature to 700 °C. Figure 5 shows the neutron diffraction patterns (90° detector bank) for the *x* = 0.2 composition. As can be seen, weak peaks corresponding to the β-phase are present in the range from 450 to 600 °C and disappear at 650 °C, leaving the pure γ-phase. On further heating to 700 °C, weak peaks of Li_2_MoO_4_ become evident. However, the appearance of this phase may be due to the extended heating at 700 °C, compared to the much shorter time spent at 650 °C. The temperature range from 450 to 600 °C, where the β-phase is observed, approximately corresponds to that where a small change in slope is seen in the thermal expansion of unit cell volume (Figure 4). However, since this change is also seen in the X-ray data for the *x* = 0.5 composition, which remains in the β-phase structure throughout, it is unlikely that the appearance of small amounts of the β-phase in the *x* = 0.2 composition accounts for the observed trend in unit cell volume expansion.

In all compositions, the apparent transition at around 400 °C leads to a larger volume at higher temperatures than expected from a linear extrapolation of the low-temperature values. This would be consistent, for example, with a small reduction of Mo^6+^ to Mo^5+^, accompanied by the creation of oxygen vacancies. However, if this was the case, then one would expect this change to be most significant in the higher *x*-value compositions where the Mo concentration is highest, but similar levels of deviation are seen throughout the compositional range. While the observed transition could be associated with a change in defect structure, as previously suggested for the related Li_3_Zn_0.5_GeO_4_ system,^17^ its presence at *x* = 0.5 indicates that either this attribution is wrong, or that the local structure of the β-phase differs from the average picture obtained from the Rietveld analysis.

### 3.3 Pellet Morphology and Electrical Behavior

**Figure 6:**
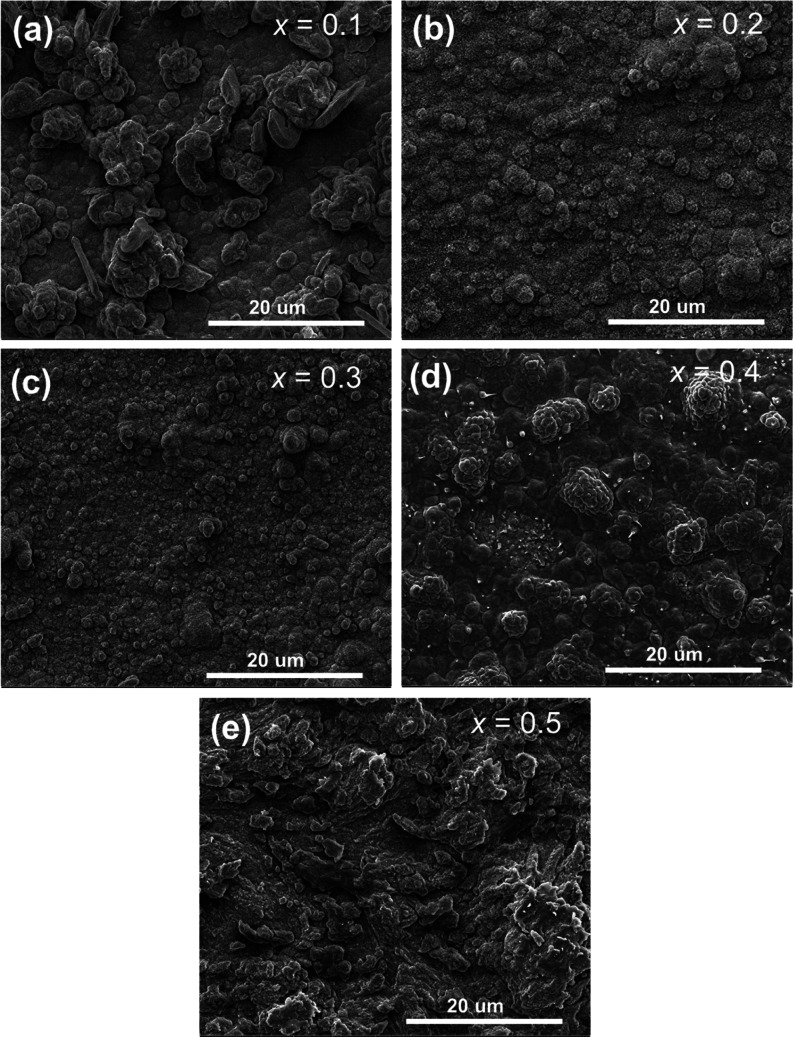
SEM surface images for SPS pellets of (a) *x* = 0.1, (b) *x* = 0.2, (c) *x* = 0.3, (d) *x* = 0.4, and (e) *x* = 0.5 compositions in the Li_4–2*x*_Ge_1–*x*_Mo_*x*_O_4_ system.

Pellets of the *x* = 0.0, 0.1, 0.2, 0.3, 0.4, 0.5, and 1.0 compositions in the Li_4–2*x*_Ge_1–*x*_Mo_*x*_O_4_ system were prepared by SPS for impedance analysis. All pellets for these compositions had relative densities of over 95% of the theoretical value, with the relative densities of the intermediate compositions of 0.1 ≤ *x* ≤ 0.5 over 98% (Table S2). Surface and fracture SEM images for Li_4–2*x*_Ge_1–*x*_Mo_*x*_O_4_ SPS pellets after annealing are shown in Figures 6 and S9, respectively. The images confirm good densification for all compositions, and the microstructure reveals particle aggregates of *ca.* 2 μm in size, made up of smaller crystallites around 300 nm in size, for all compositions. The maintenance of small crystallite size in the SPS method is one of the benefits of the short sintering process and avoids commonly encountered problems associated with oversintering in conventional sintering methods. The XRD patterns of the SPS-sintered pellets are shown in Figure S10 and reveal high crystallinity and high purity, with no evidence of significant differences in phase content from the unsintered powders.

**Figure 7:**
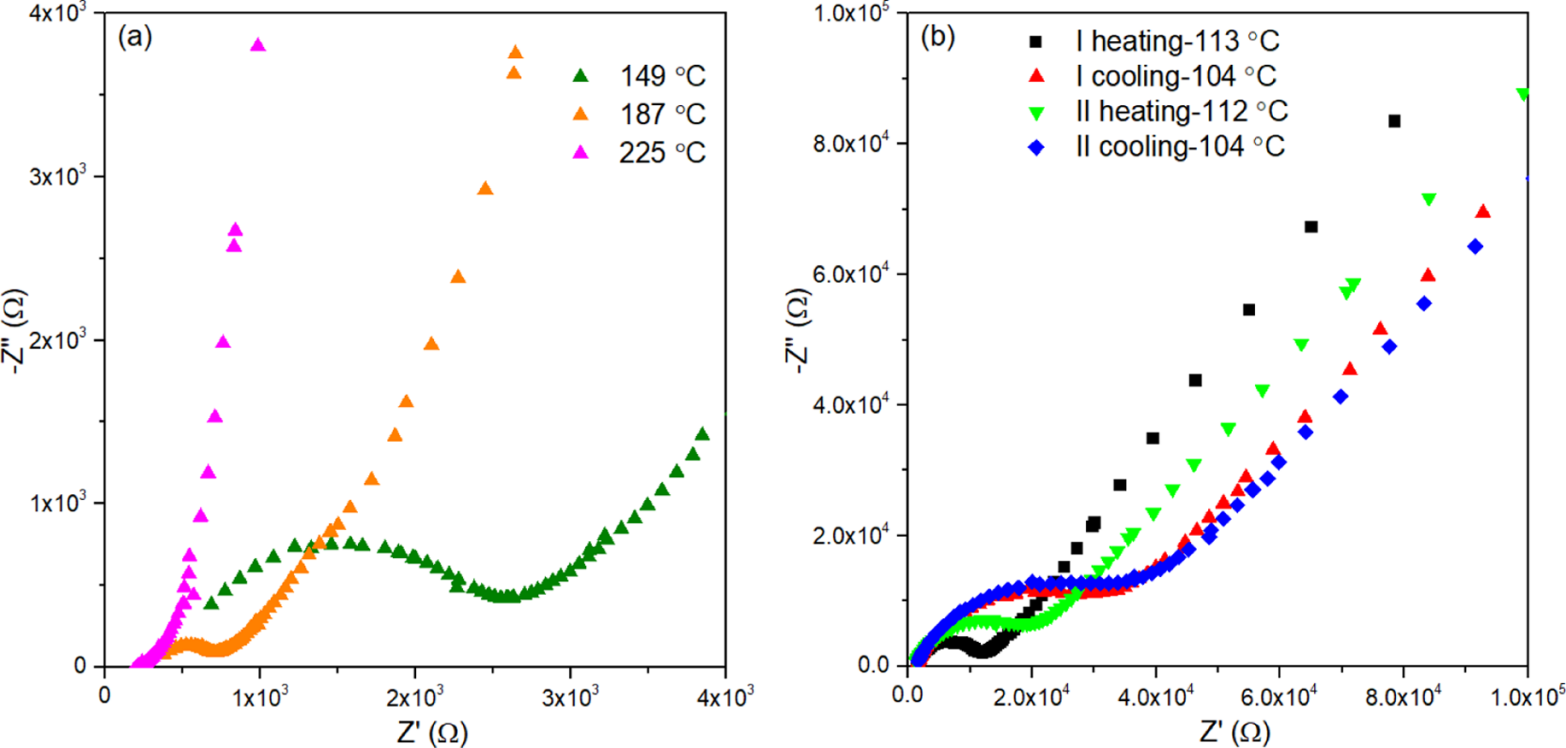
Nyquist plots for Li_3.6_Ge_0.8_Mo_0.2_O_4_ at (a) selected temperatures during the first heating run and (b) around 100 °C over consecutive heating and cooling runs.

AC impedance spectroscopy was employed to study the electrical response of the studied compositions in the Li_4–2*x*_Ge_1–*x*_Mo_*x*_O_4_ system. Figure 7a shows Nyquist plots for Li_3.6_Ge_0.8_Mo_0.2_O_4_ at selected temperatures during the first heating run. As can be seen, the spectra show a capacitive semicircle at low temperatures, with a nonzero high-frequency intercept with the real axis, and an inclined capacitive spike associated with the blocking electrode at low frequencies. With increasing temperature, the semicircle gets smaller, consistent with increasing conductivity with increasing temperature. Figure 7b shows the Nyquist plots for Li_3.6_Ge_0.8_Mo_0.2_O_4_ at *ca.* 110 °C during two successive heating and cooling runs. Interestingly, compared to the first heating run, the semicircle broadens to higher resistance values in the first cooling run. A similar variation occurs in the second heating–cooling cycle. In all cases, the high-frequency intercept with the real axis was similar.

**Figure 8:**
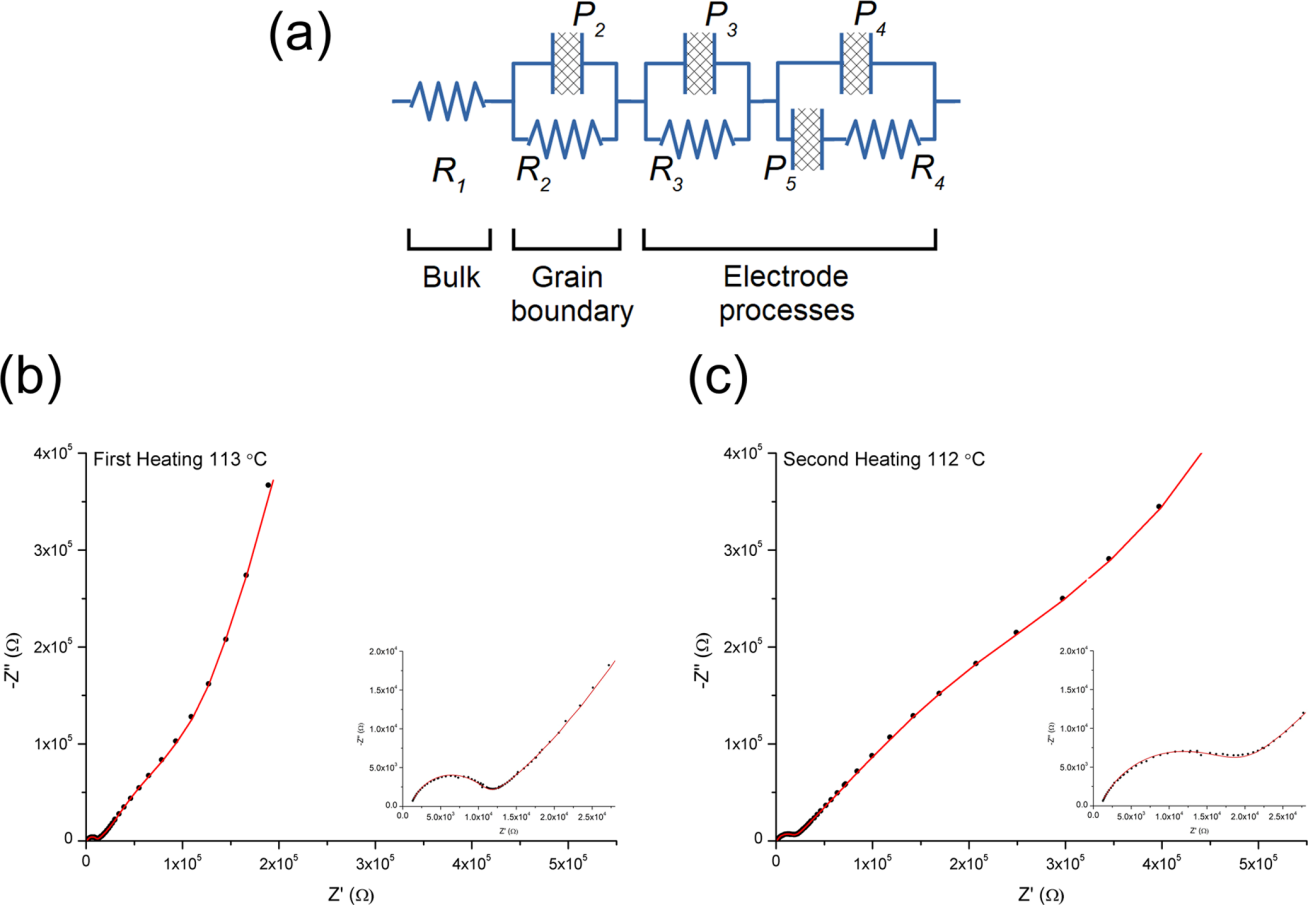
(a) Equivalent circuit, (b,c) fitted Nyquist plots showing observed (points) and calculated (line) spectra, with magnification near the origin inset, for Li_3.6_Ge_0.8_Mo_0.2_O_4_ at *ca.* 112 °C in the (b) first and (c) second heating runs.

To understand the intrinsic electrical behavior, the optimized equivalent circuit depicted in Figure 8a was used to carefully fit the impedance spectra. Figure 8b,c shows the fitted spectra at *ca.* 112 °C during the first and second heating runs with fitted equivalent circuit parameters summarized in Table S4. As can be seen in Figure 8b,c, good fitting is achieved over the whole frequency range. The resistance *R*_1_ corresponds to the intercept with the real axis, constant-phase element *P*_2_ and resistance *R*_2_ mainly contribute to the semicircle, and the values *R*_3_ and *P*_3_, associated with the Warburg impedance, and *R*_4_, *P*_4_, and *P*_5_ mainly contribute to the nonlinear tail. Based on the empirical capacitance values and the likely responsible electrical phenomena,^23^*P*_2_ with a value in the order of 10^–9^ F and *R*_2_ can be attributed to the grain boundary dispersion. However, noting the thermal behavior of the associated semicircle, *P*_2_ may well incorporate part of the complex polarization processes that occur at the electrode–electrolyte interface, with *R*_3_, *P*_3_, *R*_4_, *P*_4_, and *P*_5_, more closely associated with the electrode itself. *R*_1_ can be regarded as the bulk resistance of the Li_3.6_Ge_0.8_Mo_0.2_O_4_ sample and can be seen to decrease slightly between the first and second heating runs. It should be noted that the tail-related parameters may cover complex processes, like diffusion-controlled adsorption or reaction at the electrode, which would require especially designed experiments to characterize them.

**Figure 9:**
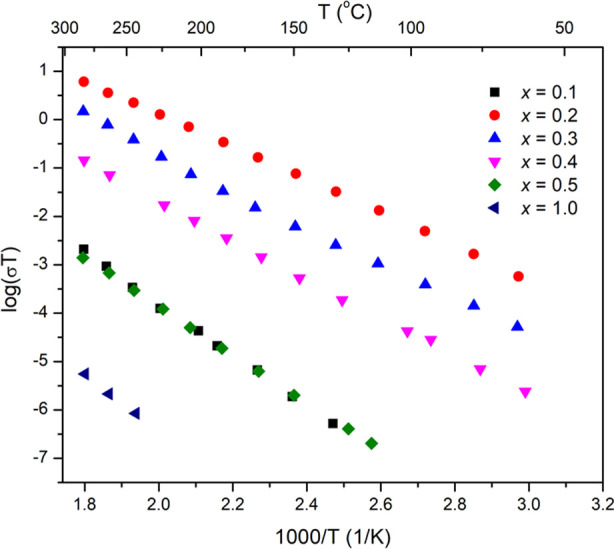
Arrhenius plots of total conductivity (second heating run) for studied compositions in the Li_4–2*x*_Ge_1–*x*_Mo_*x*_O_4_ system.

Figure 9 shows the Arrhenius plots of total conductivity for the studied compositions in the Li_4–2*x*_Ge_1–*x*_Mo_*x*_O_4_ system. All samples showed reasonable reproducibility after the first heating run, and therefore, data for the second heating run are shown. For all the compositions except for *x* = 0.3, a linear Arrhenius plot is seen over the entire studied temperature range. Only for the *x* = 0.3 composition is a change in activation energy seen, indicative of a transition at around 200 °C. Table 1 summarizes the conductivities at selected temperatures, along with activation energies for the studied compositions [for *x* = 0.3, activation energies in the low (Δ*E*_LT_) and high-temperature (Δ*E*_HT_) regions are included]. High activation energies (>1 eV) and low conductivities are seen for the two end members, Li_4_GeO_4_ and Li_2_MoO_4_. Among all the compositions studied, the highest conductivity and lowest activation energy are shown by the *x* = 0.2 composition, with conductivity values of 1.11 × 10^–7^ S cm^–1^ at room temperature and 5.02 × 10^–3^ S cm^–1^ at 250 °C, and an activation energy of 0.67 eV. While the *x* = 0.2 composition shows appreciable conductivity at 250 °C, the room temperature value is low compared to other well-studied oxide systems, such as the Li_0.34_La_0.56_TiO_3_ with the perovskite structure,^24^ Li_1.3_Al_0.3_Ti_1.7_(PO_4_)_3_ with the NASICON structure,^25^ and Li_6.55_Ga_0.15_La_3_Zr_2_O_12_ with the garnet structure,^26^ which limits its practical use.

**Table 1:** Activation Energies at Low Temperature (Δ*E*_LT_) and High Temperature (Δ*E*_HT_) and Total Conductivities (σ_temp°C_) at Selected Temperatures for Compositions in the Li_4–2*x*_Ge_1–*x*_Mo_*x*_O_4_ System^a^

*x*	Δ*E*_LT_ (eV)	Δ*E*_HT_ (eV)	σ_25°C_ (S cm^–1^)	σ_100°C_ (S cm^–1^)	σ_150°C_ (S cm^–1^)	σ_250°C_ (S cm^–1^)
0.0	1.05					8.08 × 10^–8^
0.1	1.05		3.53 × 10^–14^	1.05 × 10^–10^	4.40 × 10^–9^	8.79 × 10^–7^
0.2	0.67		1.11 × 10^–7^	1.73 × 10^–5^	1.81 × 10^–4^	5.02 × 10^–3^
0.3	0.70	0.89	7.54 × 10^–9^	1.43 × 10^–6^	1.65 × 10^–5^	8.83 × 10^–4^
0.4	0.79		2.73 × 10^–10^	1.05 × 10^–7^	1.68 × 10^–6^	8.55 × 10^–5^
0.5	0.98		9.01 × 10^–14^	1.57 × 10^–10^	5.14 × 10^–9^	7.20 × 10^–7^
1.0	1.18					2.28 × 10^–9^

a Data correspond to the second heating run. Estimated errors are ±1%. Values at 25 °C were obtained through extrapolation.

### 3.4 Average Structure Analysis

**Figure 10:**
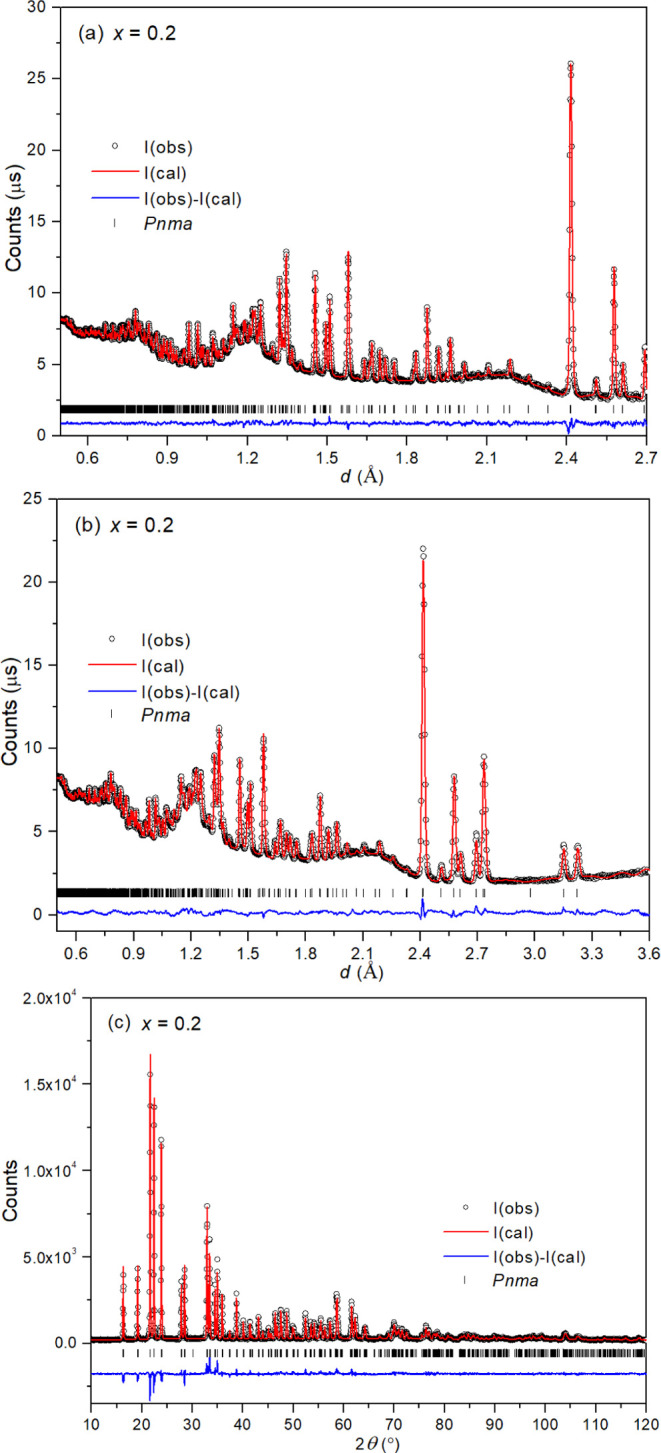
Fitted diffraction profiles for Li_3.6_Ge_0.8_Mo_0.2_O_4_ (*x* = 0.2) showing fits to (a) neutron back-scattering, (b) neutron 90°, and (c) X-ray data. Observed (circles), calculated (line), and difference (lower) profiles are shown, with reflection positions indicated by markers.

To examine the lithium-ion distribution and local defect structure, which determine the lithium-ion conductivity in the Li_4–2*x*_Ge_1–*x*_Mo_*x*_O_4_ system, the intermediate composition *x* = 0.2 in the γ-phase structure, *x* = 0.5 in the β-phase structure and the two end members Li_4_GeO_4_ and Li_2_MoO_4_ were investigated using a combined neutron and X-ray diffraction approach. The fitted diffraction profiles are shown in Figures 10 and S11–S13, with crystal and refinement parameters given in Table S5, refined structural parameters in Tables S6–S9, and significant contacts and angles in Tables S10–S13.

The refined structural parameters in Tables S6, S9, and S8 confirm that the end members, Li_4_GeO_4_ and Li_2_MoO_4_, and β-Li_3_Ge_0.5_Mo_0.5_O_4_, all have distinct structures. All show full occupancy of their respective Li tetrahedral sites (Li1 and Li2) in their structures with no occupation of interstitial tetrahedral or octahedral sites. There is good agreement between the refined structural parameters for Li_2_MoO_4_ and those presented by Kolitsch,^19^ from single-crystal X-ray diffraction data. The refined structure of Li_4_GeO_4_ is in good agreement with that determined from single-crystal X-ray data presented by Hofmann and Hoppe.^27^ In the present case, the use of neutron diffraction has enabled greater accuracy in the Li positions.

**Figure 11:**
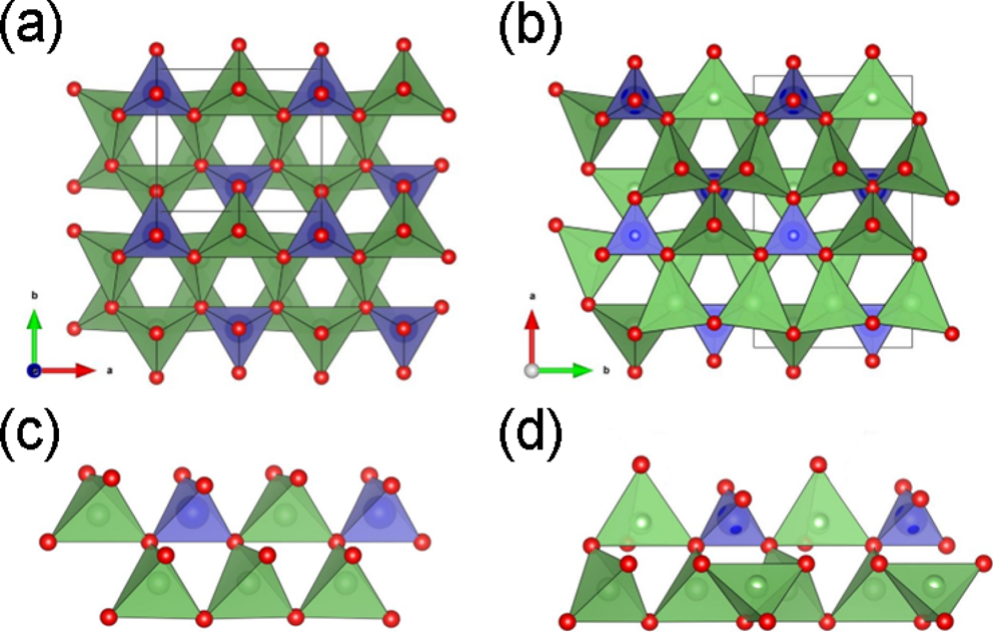
Structures of (a) β-Li_3_Ge_0.5_Mo_0.5_O_4_ and (b) γ-Li_3_PO_4_, with details of polyhedral connectivity shown in (c,d), respectively. Red spheres represent O atoms, and blue and green tetrahedra represent LiO_4_ and Ge/MoO_4_ or PO_4_, respectively.

Like the structure of the γ-phase, the structure of β-Li_3_Ge_0.5_Mo_0.5_O_4_ is based on a distorted hexagonal close-packed (hcp) array of oxide ions, with lithium, germanium, and molybdenum occupying half the tetrahedral sites but with no occupancy of interstitial octahedral sites. The most important difference between the γ- and β-phases lies in which of the tetrahedral sites in their respective hcp lattices are occupied. In the β-phase, all the tetrahedra have the same orientation and only corner-sharing occurs between adjacent tetrahedra (Figure 11a,c) as in the wurtzite structure. In contrast, in the γ-phase, edge sharing between adjacent LiO_4_ tetrahedra occurs, resulting in a structural unit consisting of three edge-sharing LiO_4_ tetrahedra which corner-shares with an MO_4_ (M = Ge, Mo in the present case) tetrahedron (Figure 11d). These moieties corner-share to give the structural framework of the γ-phase (Figure 11b).

Examining the significant contact distances and angles in Tables S10–S13, it can be seen that the average Ge/Mo–O bond length shows only a very small increase as Ge^4+^ is replaced by the larger Mo^6+^ cation in the Li_4_GeO_4_–Li_2_MoO_4_ system (1.76 to 1.77 Å). The average Li–O distance is highest in the *x* = 0.2 composition at 2.06 Å but in other compositions is in the range of 1.97 to 1.98 Å. The high value for the *x* = 0.2 composition is due to the presence of nonframework octahedral (Li3 and Li4) and displaced framework (Li1a and Li2a) Li^+^-ions in the γ-phase structure. If only the framework Li^+^-ions (Li1 and Li2) are considered, then the average Li–O value of 1.98 Å is comparable to those in the other compositions. Interestingly, in the *x* = 0.2 composition, the average Li1–O distance (1.99 Å) is larger than that for Li2–O (1.97 Å), indicating that the Li1 tetrahedral site is larger.

**Figure 12:**
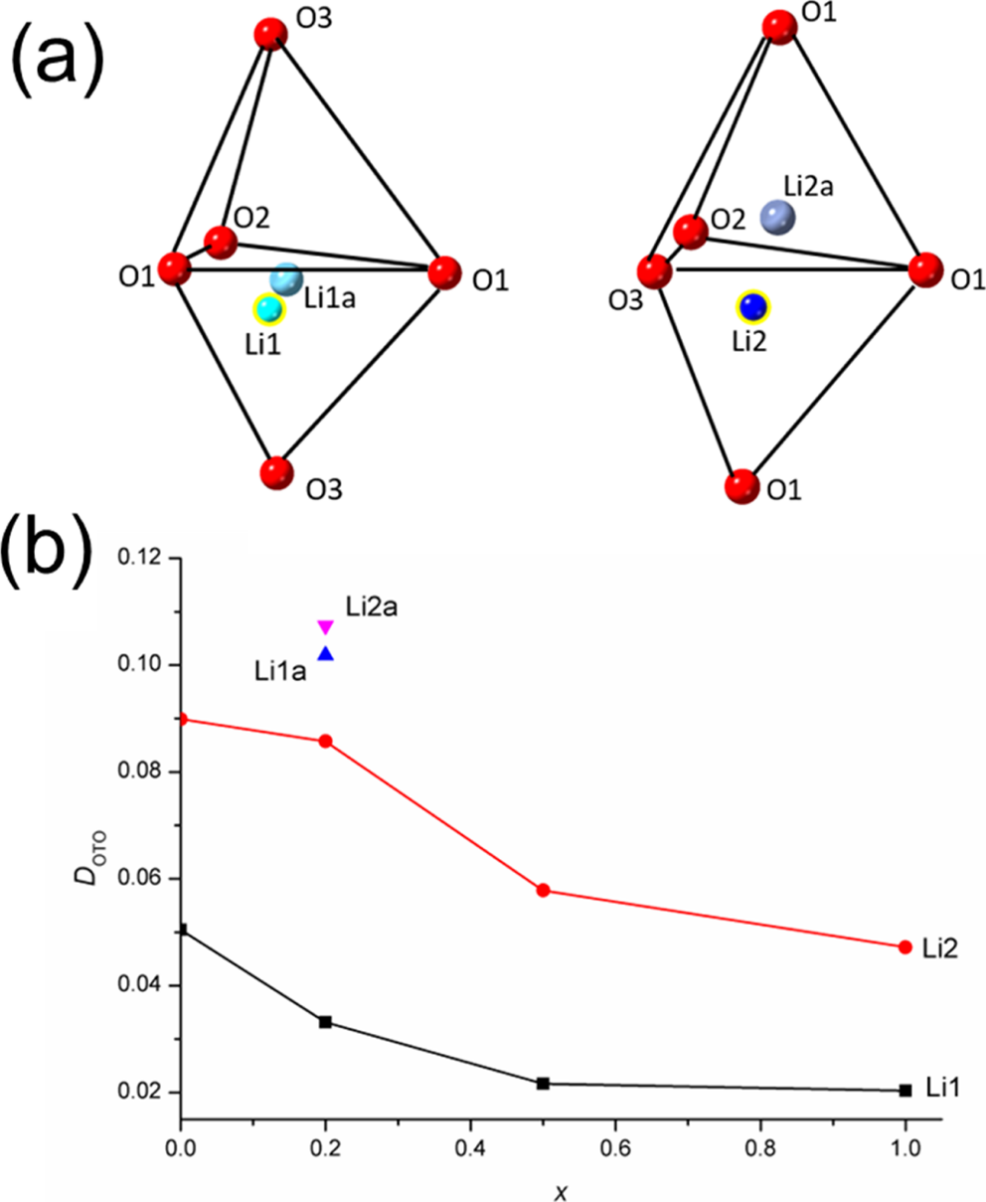
(a) Li^+^-ion positions in tetrahedral Li sites in Li_3.6_Ge_0.8_Mo_0.2_O_4_ and (b) compositional variation of angular tetrahedral distortion index, *D*_OTO_, in the Li_4–2*x*_Ge_1–*x*_Mo_*x*_O_4_ system.

Unlike Li_4_GeO_4_, Li_2_MoO_4_ and β-Li_3_Ge_0.5_Mo_0.5_O_4_, Li_3.6_Ge_0.8_Mo_0.2_O_4_ (*x* = 0.2) shows occupation of octahedral sites in the hcp lattice. The presence of Li^+^-ions in these sites leads to displacement of Li^+^ ions in tetrahedral sites (Li1 and Li2) toward neighboring interstitial tetrahedral sites parallel to the *c*-axis direction as a result of the repulsive forces between Li^+^-ions in the neighboring octahedral sites, Li3 and Li4. In Li_3.6_Ge_0.8_Mo_0.2_O_4_, both Li2 → Li2a and Li1 → Li1a displacements are relatively large compared to other LISICON-structured systems.^28,29^Figure 12a illustrates the Li^+^-ion positions in tetrahedral Li sites for Li_3.6_Ge_0.8_Mo_0.2_O_4_. As seen in Figure 12a and Table 2, Li1a is displaced by a distance of 0.625 Å from Li1, although still sitting within the same tetrahedron, while Li2a is displaced by a distance of 1.105 Å away from Li2, putting the Li2a ions in the neighboring interstitial tetrahedral site. The Li1a location is near the shared face between the occupied and unoccupied tetrahedra, but the distance to the furthest oxygen in the unoccupied tetrahedron (O3) is 2.927 Å, meaning Li1a cannot be considered to be 5-coordinate. It is noteworthy that the thermal parameters of Li3 and Li4 are several times higher than those for the Li1/Li1a and Li2/Li2a pairs, indicating significant positional disorder in these interstitial sites.

**Table 2:** (a) Li...Li Intersite Contact Distances and (b) Site Occupancy Ratios for Li_3.6_Ge_0.8_Mo_0.2_O_4_ from Conventional Rietveld Analysis

(a)			
ion pair	distance (Å)	ion pair	distance (Å)
Li1...Li1a	0.63(2)	Li1...Li2	2.60(1) × 2
Li1...Li2′	2.79(2) × 2	Li1...Li3	2.35(4)
Li1...Li4	1.84(1) × 2	Li1a...Li2	2.614(8)
Li1a...Li2a	2.64(2) × 2	Li1a...Li3	1.75(3)
Li1a...Li4	2.201(9) × 2	Li2...Li2a	1.11(2)
Li2...Li3	2.25(2)	Li2...Li3′	2.50(2)
Li2...Li4	1.970(4)	Li2a...Li3	1.51(2)
Li2a...Li3′	2.88(2)	Li2a...Li4	2.76(2)
Li3...Li4	3.60(2)	Li3...Li4′	3.64(2)

(b)			
ion pair	ratio	ion pair	ratio
Li1a/Li3	1.60	Li2a/Li3	0.90
Li1a/Li4	3.72	Li2a/Li4	2.09
Li1a/(Li3 + Li4)	1.12	Li2a/(Li3 + Li4)	0.63
Li3/Li4	2.33		

It is helpful to look at the distortion of the framework LiO_4_ tetrahedral sites. The angular tetrahedral distortion index, *D*_OTO_, as defined by Baur,^30^ is given by
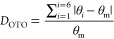
1where θ_*i*_ and θ_m_ are the *i*th and mean O–Li–O angles, respectively. The compositional variation of *D*_OTO_ for the framework LiO_4_ tetrahedra is shown in Figure 12b. For both types of framework tetrahedron, *D*_OTO_ shows a general decreasing trend with increasing Mo content. The tetrahedron around Li2 is considerably more distorted than that around Li1. This is likely related to the fact that the Li1 site is larger, as discussed above. Unsurprisingly, the distortions in the tetrahedral coordination environments of the displaced cations in the *x* = 0.2 composition are very much larger than those for the corresponding framework ions Li1 and Li2. The relative sizes of the two LiO_4_ framework sites and the resulting higher distortion of the tetrahedral coordination of the Li2 ion may explain the extent of displacement of the Li2a site away from Li2 compared to that between Li1a and Li1.

The high ionic conductivity in Li_3.6_Ge_0.8_Mo_0.2_O_4_ is related to its local structure. Using the structural data, it is possible to propose individual defects in Li_3.6_Ge_0.8_Mo_0.2_O_4_. Table 2 contains details of the Li...Li contacts and site occupancy ratios, including short distances that preclude the simultaneous occupancy of sites.

From the intersite ratios in Table 2, the Li3/Li2a ratio is approximately 1, indicating that the presence of a Li^+^-ion on the Li3 site causes displacement of one of the neighboring Li2 ions (at a distance of 2.252 Å) into the Li2a site. The Li2a/Li4 ratio is approximately 2, indicating that the Li^+^-ions in both the Li2 sites that share faces with the Li4 site, with a Li2...Li4 distance of 1.970 Å, are displaced into the neighboring Li2a sites. The displacement of Li1a seems to be caused by the presence of Li^+^-ions in both Li3 and Li4 sites, with the Li1a/(Li3 + Li4) ratio approximating to 1. The Li3/Li4 ratio is approximately 2, suggesting clustering of these individual defects.

**Figure 13:**
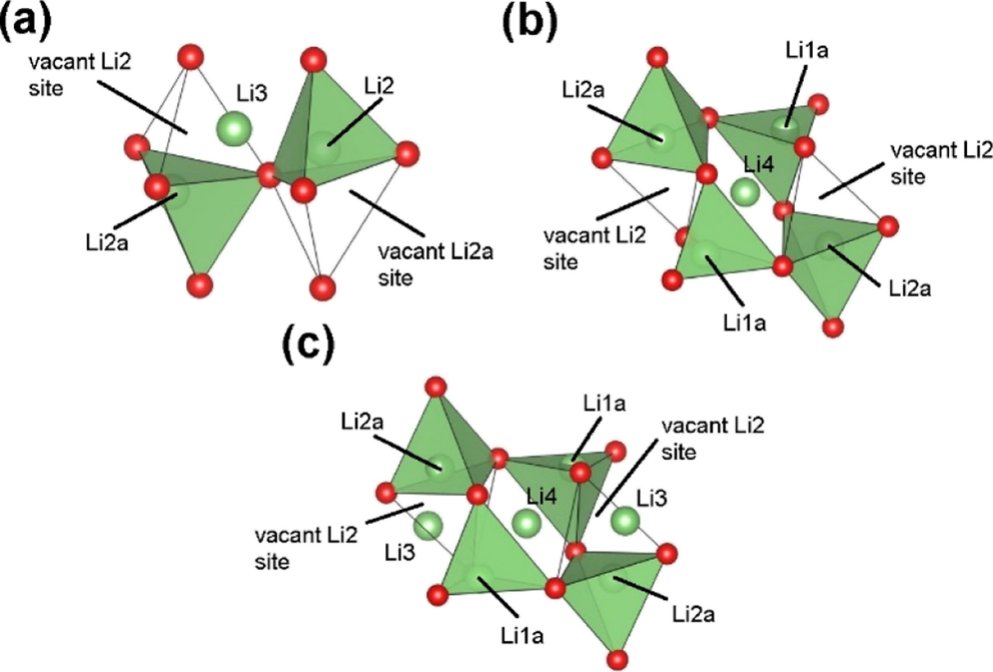
Proposed defect clusters in Li_3.6_Ge_0.8_Mo_0.2_O_4_: (a) type I, (b) type IIa, and (c) type III defect clusters.

The proposed defects are summarized in Figure 13 and are similar to those we have previously proposed in the Li_3_Zn_0.5_GeO_4_ and Li_3.5_Ge_0.5_V_0.5_O_4_ systems.^10,28^ The simplest of these (Figure 13a) involves Li^+^-ions in the Li3 site which displace Li^+^-ions in a neighboring Li2 site into an empty tetrahedral site (Li2a). This defect is analogous to the type I defect cluster described in the structure of Li_3_Zn_0.5_GeO_4_.^10^ The second proposed cluster centers around Li4 (Figure 13b), with displacements of all the ions in the neighboring face sharing tetrahedra, *i.e.*, two Li2a and two Li1a ions. This cluster differs somewhat from those previously proposed in the Li_3_Zn_0.5_GeO_4_ and Li_3.5_Ge_0.5_V_0.5_O_4_ systems, in that all the ions in neighboring tetrahedral sites are displaced, and to clearly distinguish it, here it is designated as type IIa. The 2:1 ratio of Li3/Li4 suggests clustering of the type I and type IIa defects. The smallest cluster based on this ratio would involve three octahedral Li^+^-ions, *i.e.*, two type I defects with one type IIa in the middle (Figure 13c), which is similar to the type III cluster identified in Li_3_Zn_0.5_GeO_4_.^10,17^

## 4 Conclusions

The solid solution limit was investigated in the Li_2_MoO_4_–Li_4_GeO_4_ (Li_4–2*x*_Ge_1–*x*_Mo_*x*_O_4_) system, and it was found that γ- and β-LISICON-type structures can be formed in the compositional range 0.1 ≤ *x* ≤ 0.5 at synthesis temperatures of 650 to 850 °C. As the level of substitution increases, the β-phase becomes increasingly more stable, with the solid solution limit of the γ-phase lying between *x* = 0.3 and *x* = 0.4. A new single-phase composition, β-Li_3_Ge_0.5_Mo_0.5_O_4_, with the β-Li_3_PO_4_ structure has been isolated and shows good stability. Although this phase exhibits relatively low conductivity, due to the absence of Li^+^-ion interstitials, it might serve as the base composition for other more highly conducting solid solutions.

Rietveld analysis of combined neutron and X-ray diffraction data for the *x* = 0.2 composition revealed a γ-phase structure with two interstitial octahedral sites (Li3 and Li4) and displacement of ions in both the tetrahedral framework sites. Two basic types of defect cluster are proposed based on the intersite contact distances and occupancy ratios of the lithium sites. Both consist of ions in an interstitial octahedral site with displacement of ions in neighboring tetrahedral sites. The concentration of these basic defect clusters is relatively high in this heavily disordered system, and site ratios suggest that it is likely that there will be clustering of these simpler defect moieties into larger defect clusters, the simplest of which involves three interstitial octahedral ions and displaced ions in neighboring tetrahedral sites.

In the Li_4–2*x*_Ge_1–*x*_Mo_*x*_O_4_ system, the highest conductivity and lowest activation energy are seen in the *x* = 0.2 composition, with conductivity values of 1.11 × 10^–7^ S cm^–1^ at room temperature and 5.02 × 10^–3^ S cm^–1^ at 250 °C, and an activation energy of 0.67 eV.

## Supporting Information Available

Summary of preparation conditions, additional characterization data including refined lattice parameters, powder diffraction data, thermal variation of lattice parameters and unit cell volume, SEM images, fitted diffraction profiles, crystallographic data, contact distances and angles, and refined electrical parameters (PDF)
