# Supplementary material for: Structure and Conductivity in LISICON Analogues within the Li4GeO4–Li2MoO4 System
Source: Inorg Chem. 2023 Jul 14;62(30):11876–86. doi: 10.1021/acs.inorgchem.3c01222 (PMC10394663; doi:10.1021/acs.inorgchem.3c01222)
Supplement: Supplementary file 1 — ic3c01222_si_001.pdf [file ic3c01222_si_001.pdf]

Supplementary Information

Structure and conductivity in LISICON analogues within the Li_4GeO_4-Li_2MoO_4 system

Ludan Zhang,[a,b] Marcin Malys,[c] Jan Jamroz,[c] Franciszek Krok,[c] Wojciech Wrobel,[c] Stephen Hull,[d] Haixue Yan,[e] Isaac Abrahams* [a]

[a] Department of Chemistry, Queen Mary University of London, Mile End Road, London, E1 4NS, UK

[b] Shenzhen CAPCHEM Technology Co. Ltd., Pingshan District, Shenzhen 518118, China

[c] Faculty of Physics, Warsaw University of Technology, Koszykowa 75, 00-662 Warszawa, Poland

[d] Science and Technology Facilities Council, ISIS Facility, Rutherford Appleton Laboratory, Chilton, Didcot, Oxon OX11 OQX, UK

[e] School of Engineering and Materials Science, Queen Mary University of London, Mile End Road, London, E1 4NS, UK

*Corresponding author:

Email: i.abrahams@qmul.ac.uk

Tel: +44 207 882 3235

Table S1. Summary of preparation conditions used to prepare $Li_{4-2x}Ge_{1-x}Mo_xO_4$ samples

x	Conditions
0.1, 0.2, 0.3	650 °C for 1 h and 750 °C for 24 h
0.4	650 °C for 1 h and 650 °C/700 °C/750 °C/800 °C/850 °C/900 °C for 24 h
0.5	650 °C for 1 h and 650 °C/700 °C/725 °C/750 °C/800 °C/850 °C for 24 h
0.6	650 °C for 1 h and 750 °C for 24 h
1.0	650 °C for 25 h

Table S2. Summary of SPS sintering conditions used to prepare $Li_{4-2x}Ge_{1-x}Mo_xO_4$ pellets

x	Sintering conditions	density%
x = 0.1	750 ºC for 5 min, annealed at 750 ºC for 8 h	> 98%
x = 0.2	800 ºC for 5 min, annealed at 700 ºC for 11 h	> 99%
x = 0.3	800 ºC for 5 min, annealed at 750 ºC for 12 h	> 99%
x = 0.4	800 ºC for 5 min, annealed at 850 ºC for 20 h	> 98%
x = 0.5	600 ºC for 5 min, annealed at 600 ºC for 11 h	> 99%
x = 1.0	600 ºC for 5 min, annealed at 650 ºC for 16 h	> 95%

Table S3. Refined lattice parameters for studied compositions in the $Li_{4-2x}Ge_{1-x}Mo_xO_4$ system

composition	a (Å)	b (Å)	c (Å)	unit cell volume (Å^3)
x = 0.1	10.9278(5)	6.2458(3)	5.1635(2)	352.42(4)
x = 0.2	10.9419(3)	6.2973(2)	5.1506(1)	354.90(3)
x = 0.3	10.9323(4)	6.3374(2)	5.1458(2)	356.51(3)
x = 0.4	10.9638(3)	6.3660(2)	5.1293(1)	358.00(2)
x = 0.5	10.9746(6)	6.3674(4)	5.1236(3)	358.04(4)

Table S4. Equivalent circuit parameters for $Li_{3.6}Ge_{0.8}Mo_{0.2}O_4$ at *ca.* 112 ºC over the first and second heating runs. The elements R and P are resistors and constant phase elements, respectively, with complex capacitance defined as $C^{*}_{CPE}(\omega) = A \cdot (j\omega)^{\alpha-1}$ where A is expressed in farads, assuming that frequency ω is divided by 1 Hz, $j = \sqrt{-1}$.

**Parameters**	**First heating-113 ºC**	**Second heating-112 ºC**
	Value	Value
R_1 (Ω)	1011	899
R_2 (Ω)	9276	1.6×10^{4}
A_2 (F)	2.1×10^{-9}	6.9×10^{-9}
α_2	0.84	0.77
R_3 (Ω)	1.4×10^{5}	9.0×10^{5}
A_3 (F)	6.0×10^{-6}	1.4×10^{-6}
α_3	0.43	0.51
A_4 (F)	1.5×10^{-6}	1.9×10^{-6}
α_4	0.98	1.00
A_5 (F)	2.3×10^{-6}	2.3×10^{-6}
α_5	0.83	0.98
R_4 (Ω)	1.2×10^{5}	1.6×10^{5}

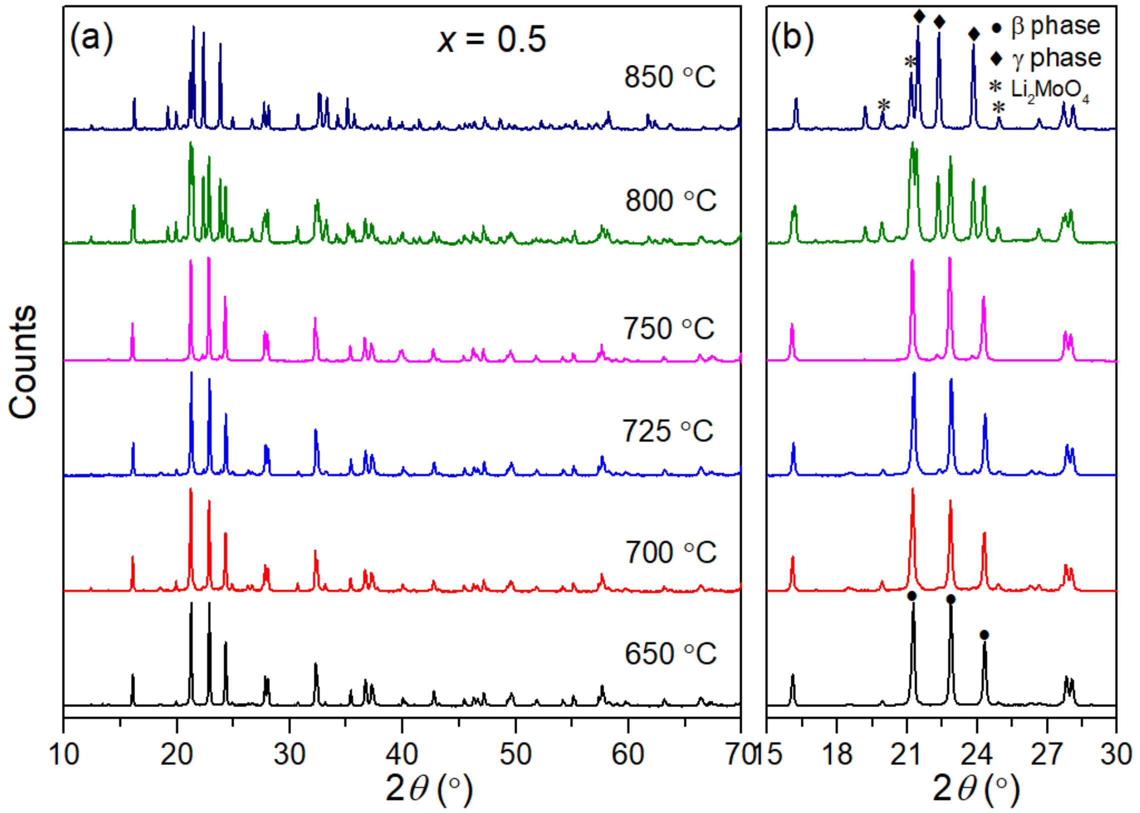

Figure S1. (a) X-ray powder diffraction patterns for the x = 0.5 composition in the $Li_{4-2x}Ge_{1-x}Mo_xO_4$ system calcined at selected temperatures for 24 h, with detail shown in (b).

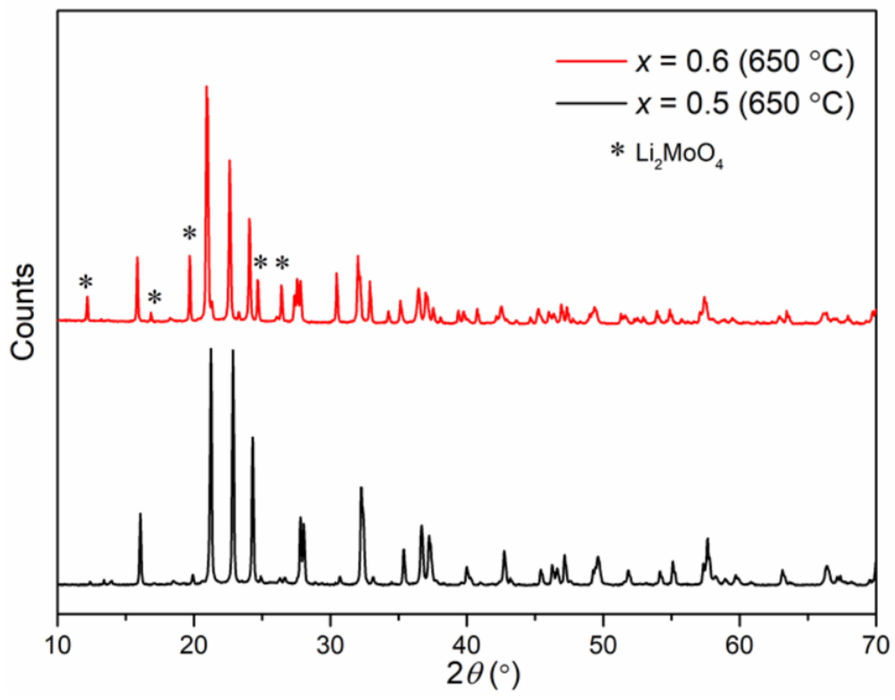

Figure S2. X-ray powder diffraction patterns for the x = 0.5 and 0.6 compositions in the $Li_{4-2x}Ge_{1-x}Mo_xO_4$ system synthesised under the same conditions for 24h

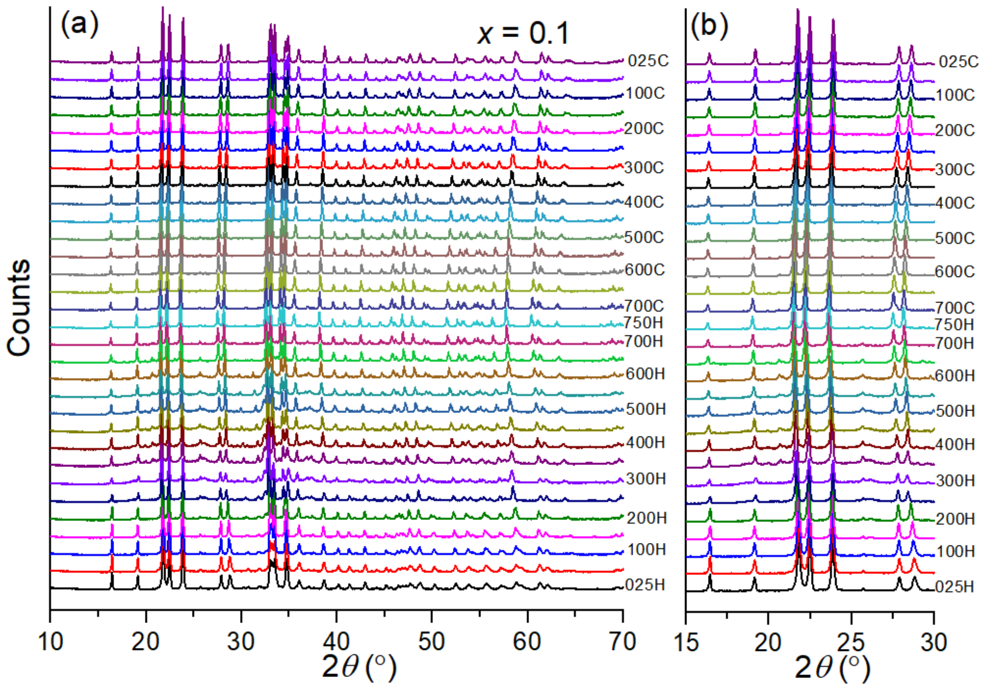

Figure S3. (a) X-ray powder diffraction patterns for $Li_{3.8}Ge_{0.9}Mo_{0.1}O_4$ (x = 0.1) on heating and cooling with detail shown in (b)

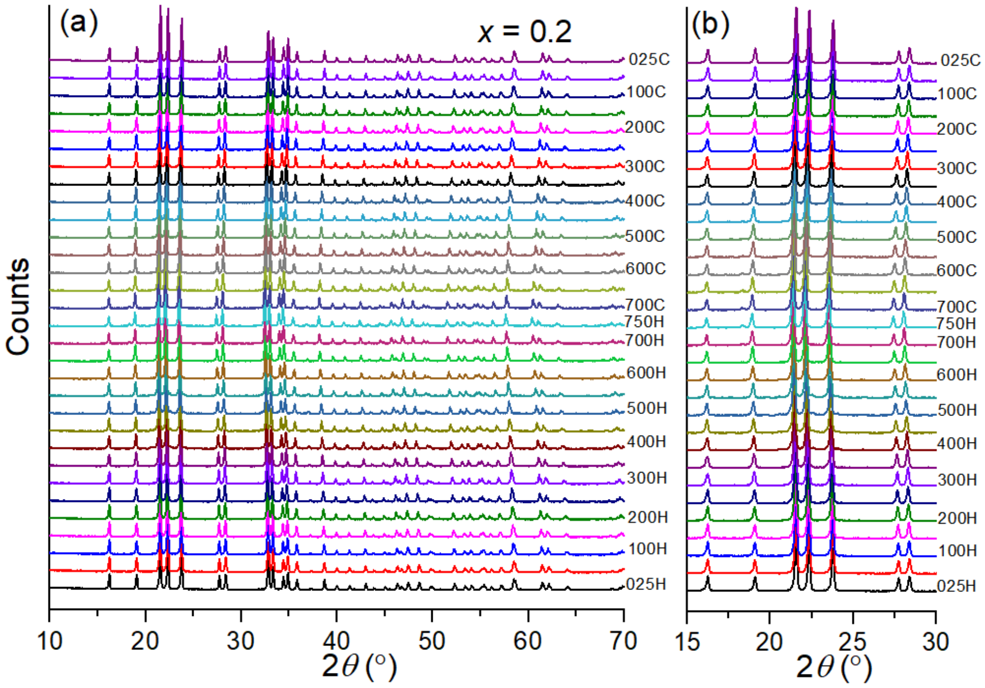

Figure S4. (a) X-ray powder diffraction patterns for $Li_{3.6}Ge_{0.8}Mo_{0.2}O_4$ (x = 0.2) on heating and cooling with detail shown in (b)

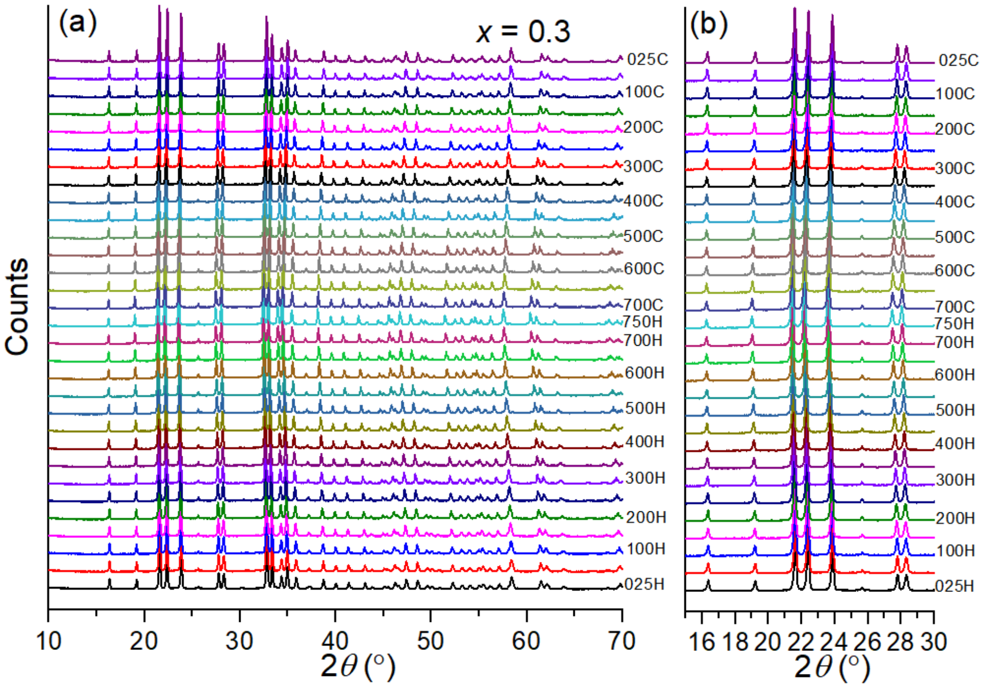

Figure S5. (a) X-ray powder diffraction patterns for $Li_{3.4}Ge_{0.7}Mo_{0.3}O_4$ (x = 0.3) on heating and cooling with detail shown in (b)

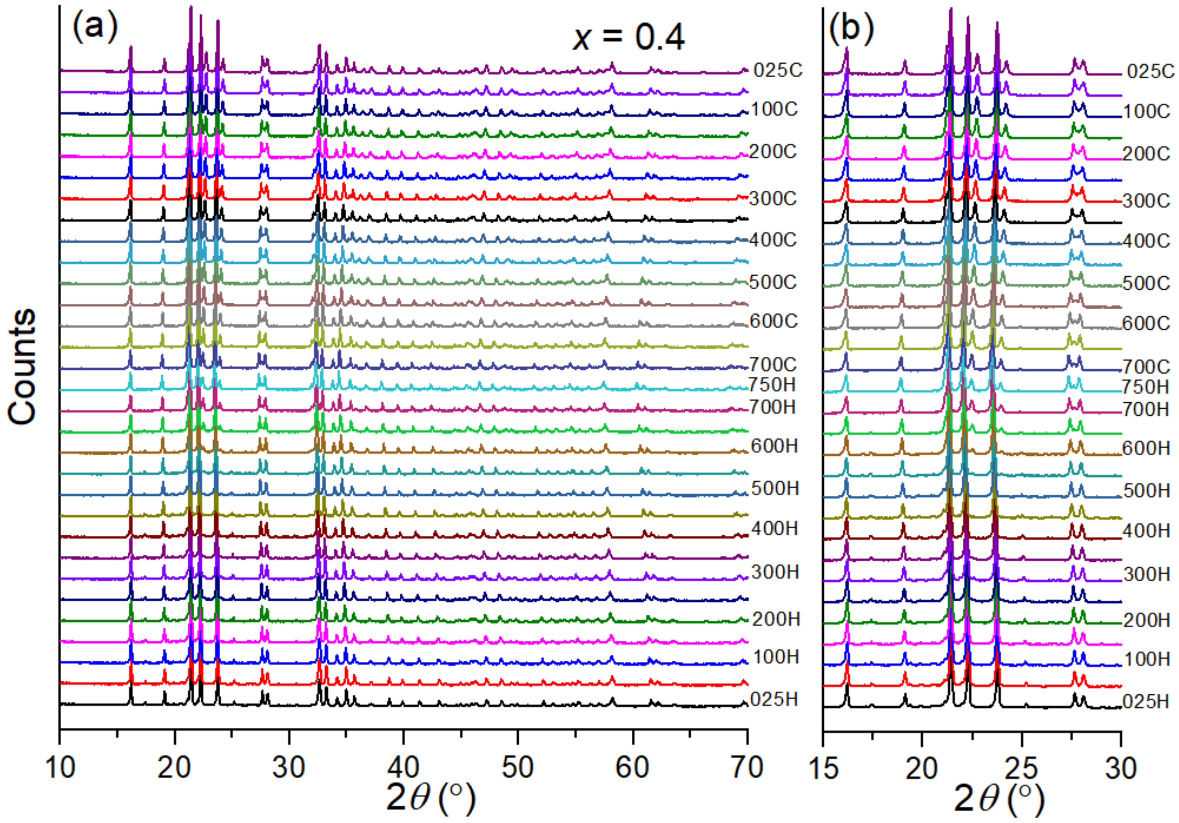

Figure S6. (a) X-ray powder diffraction patterns for $Li_{3.2}Ge_{0.6}Mo_{0.4}O_4$ (x = 0.4) on heating and cooling with detail shown in (b)

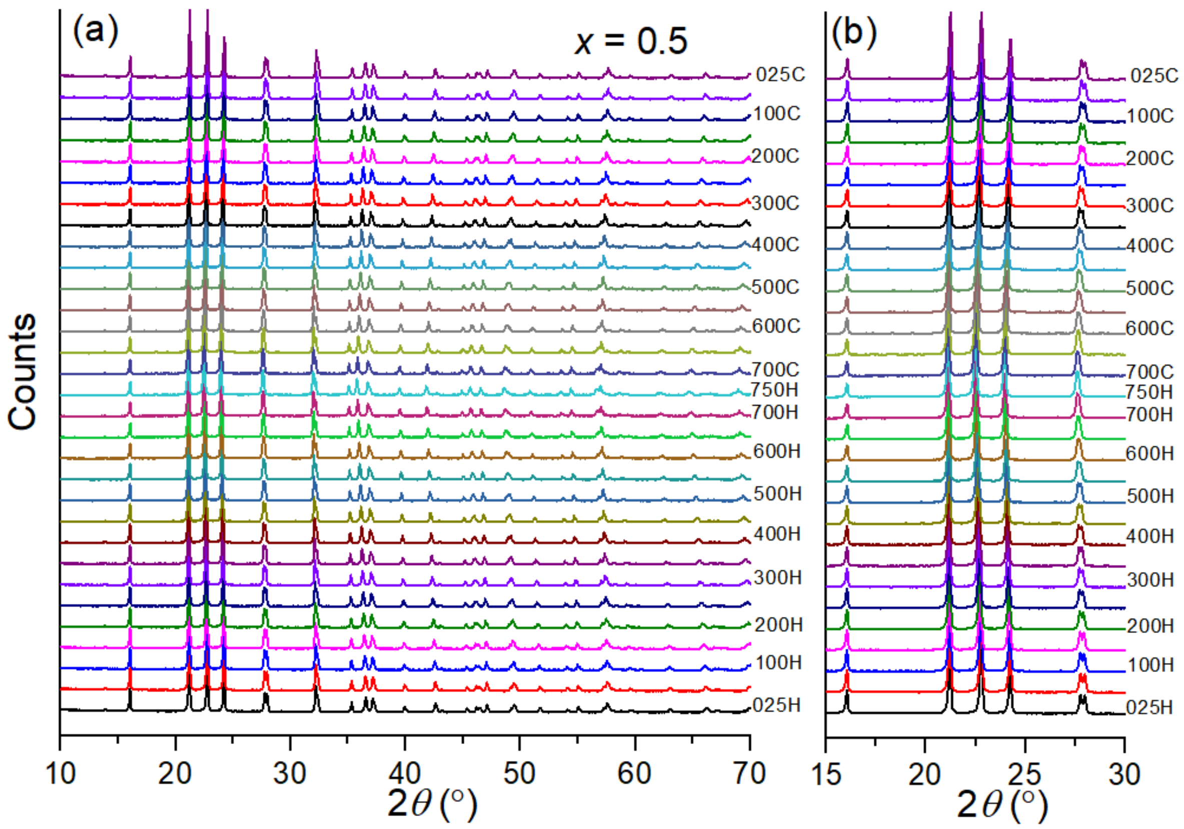

Figure S7. (a) X-ray powder diffraction patterns for $Li_3Ge_{0.5}Mo_{0.5}O_4$ (x = 0.5) on heating and cooling with detail shown in (b)

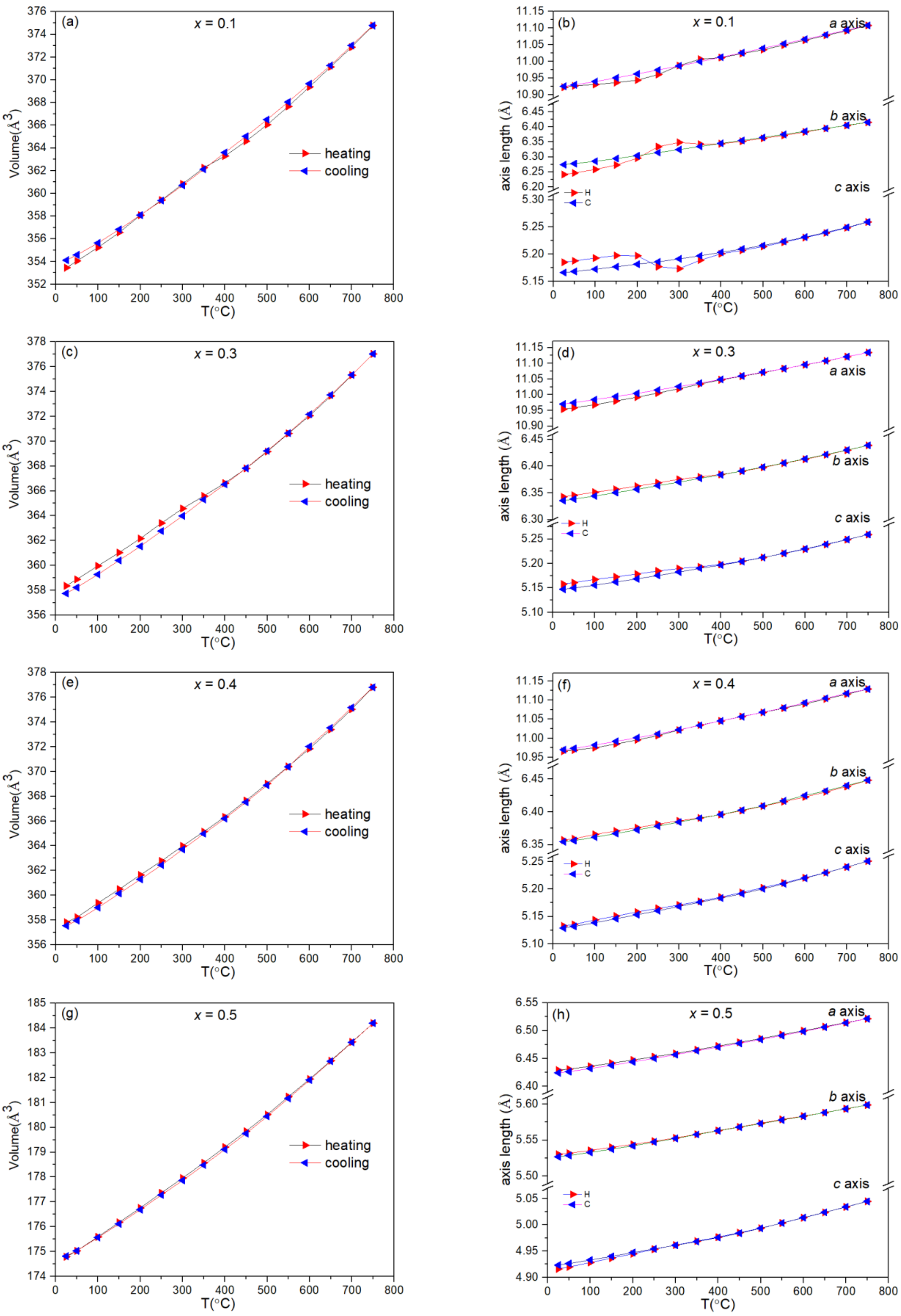

Figure S8. Thermal variation of (a, c, e, g) unit cell volume and (b, d, f, h) lattice parameters for (a-b) x = 0.1, (c-d) x = 0.3, (e-f) x = 0.4 and (g-h) x = 0.5 compositions in the $Li_{4-2x}Ge_{1-x}Mo_xO_4$ system on heating and cooling

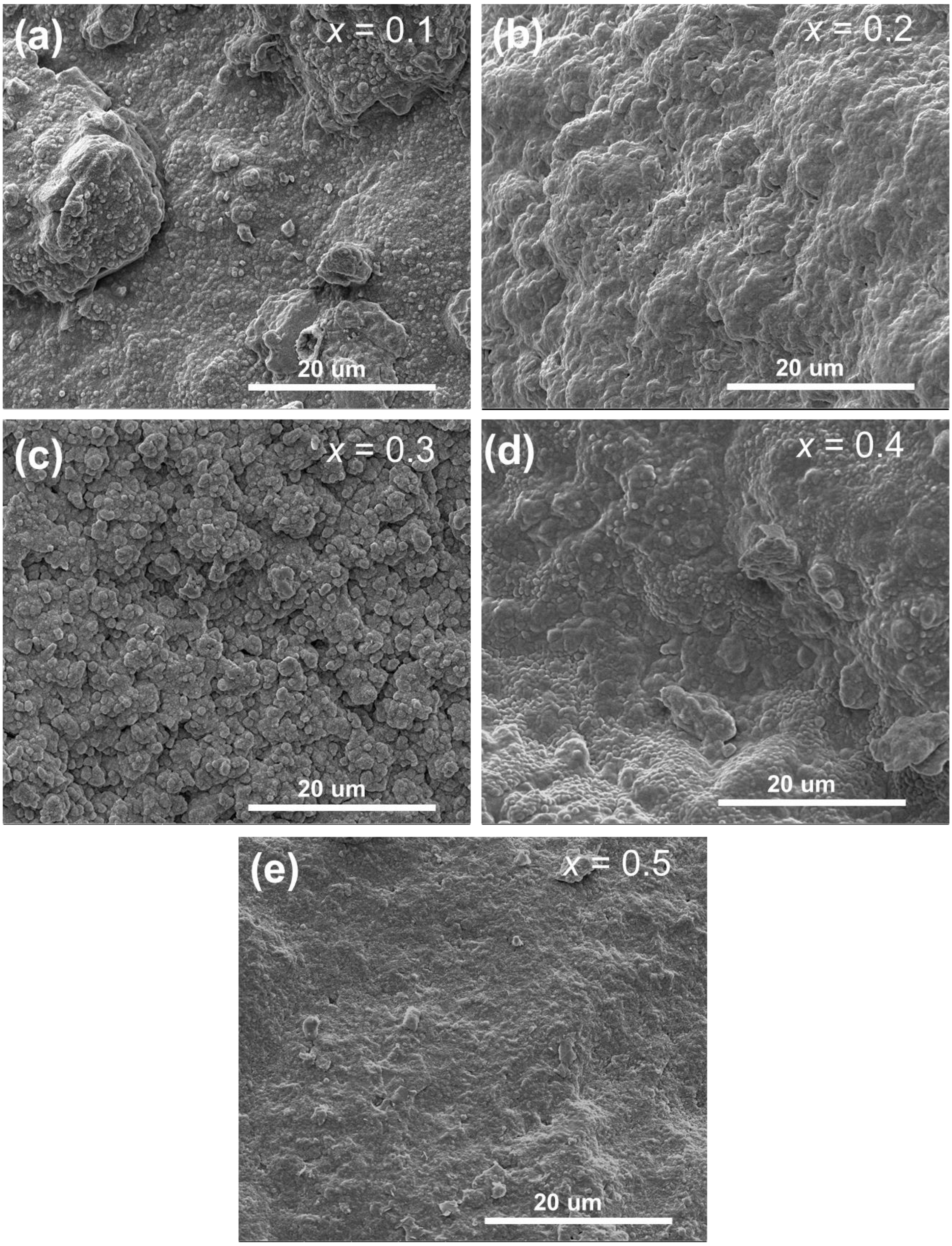

Figure S9. SEM fracture images for SPS pellets of (a) x = 0.1, (b) x = 0.2, (c) x = 0.3, (d) x = 0.4 and (e) x = 0.5 compositions in the $Li_{4-2x}Ge_{1-x}Mo_xO_4$ system

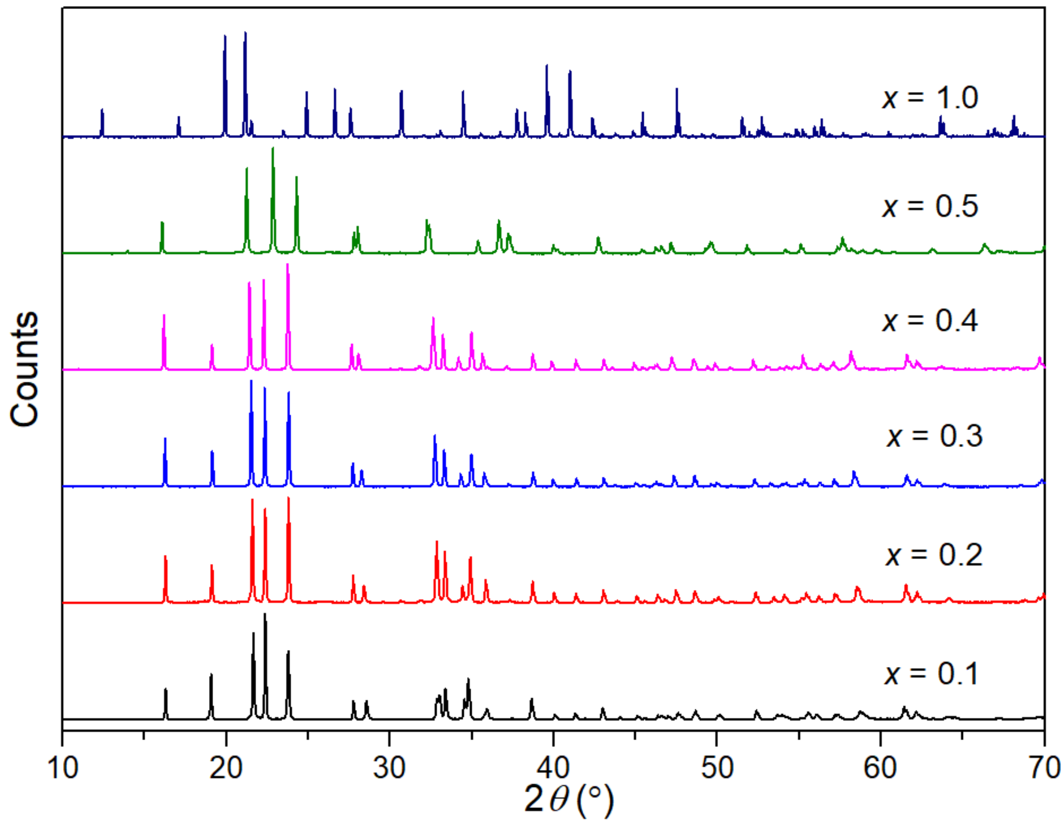

Figure S10. X-ray powder diffraction patterns for SPS-sintered pellets of x = 0.1, 0.2, 0.3, 0.4, 0.5 and 1.0 compositions in the $Li_{4-2x}Ge_{1-x}Mo_xO_4$ system

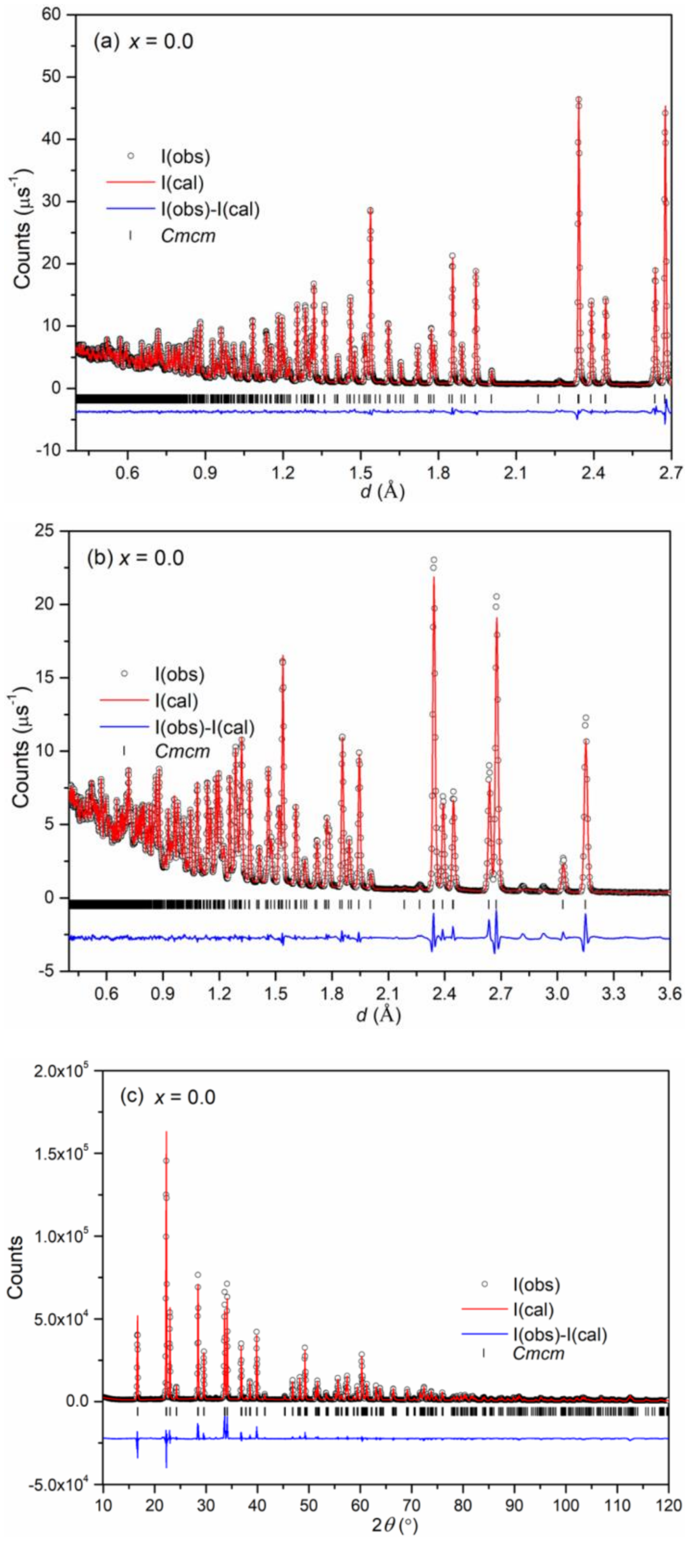

Figure S11. Fitted diffraction profiles for Li_4GeO_4 (*x* = 0.0) showing fits to (a) neutron back scattering (b) neutron 90° and (c) X-ray data. Observed (circles), calculated (line) and difference (lower) profiles are shown, with reflection positions indicated by markers

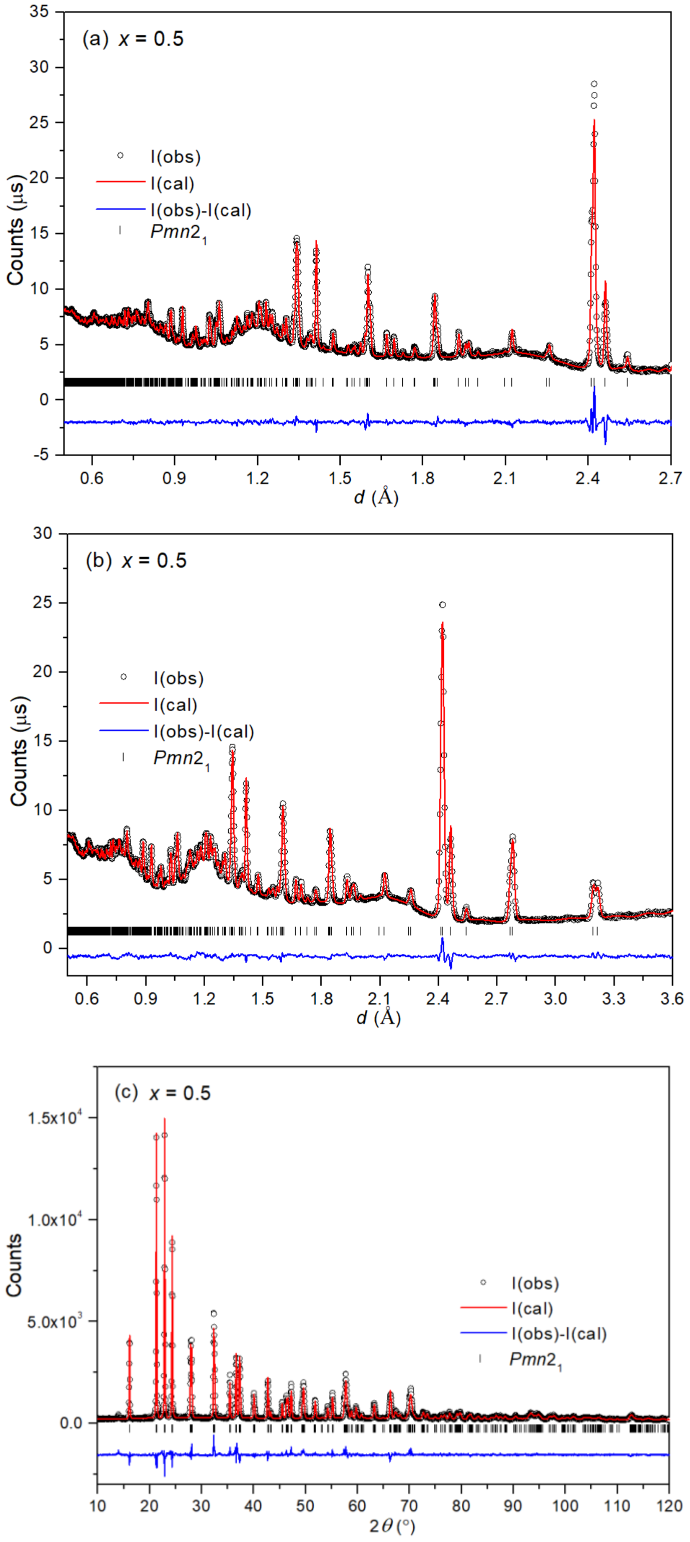

Figure S12. Fitted diffraction profiles for $Li_3Ge_{0.5}Mo_{0.5}O_4$ (x = 0.5) showing fits to (a) neutron back scattering (b) neutron 90° and (c) X-ray data. Observed (circles), calculated (line) and difference (lower) profiles are shown, with reflection positions indicated by markers

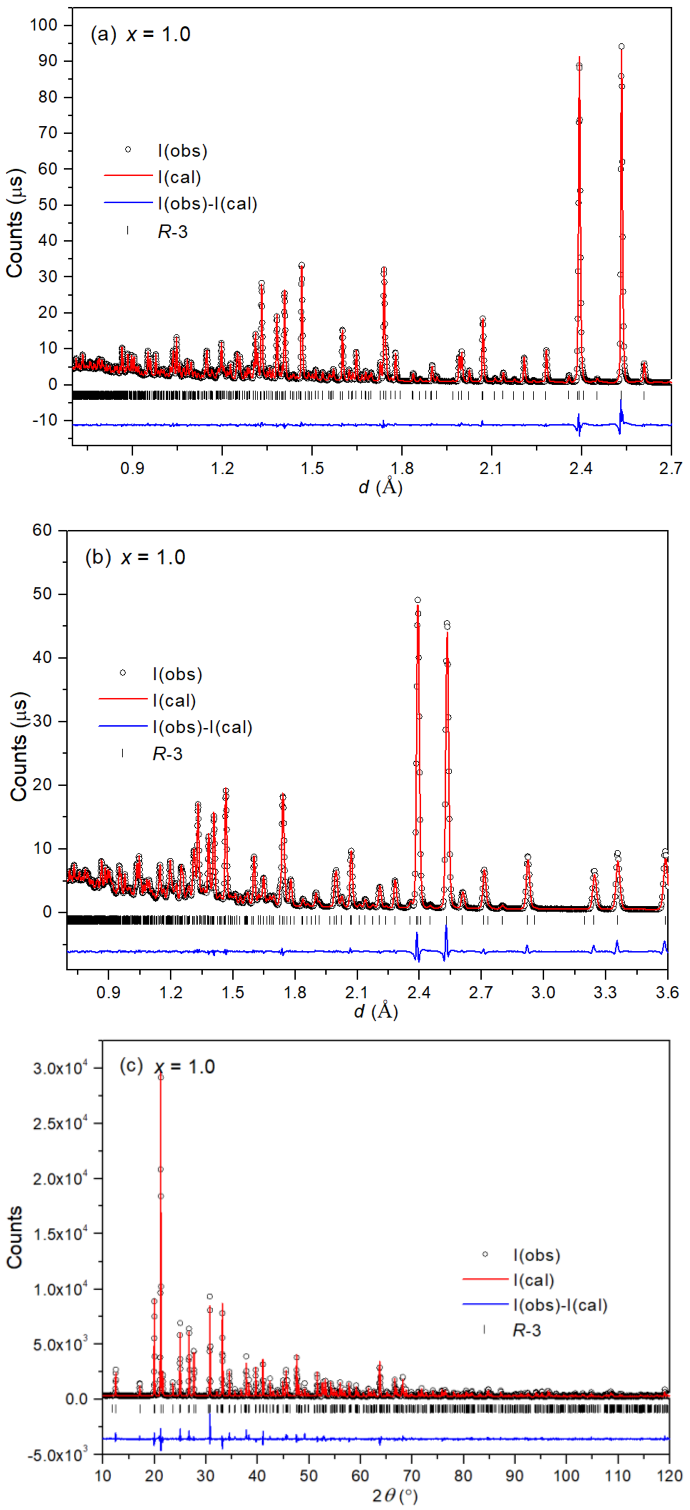

Figure S13 Fitted diffraction profiles for Li_2MoO_4 (x = 1.0) showing fits to (a) neutron back scattering (b) neutron 90° and (c) X-ray data. Observed (circles), calculated (line) and difference (lower) profiles are shown, with reflection positions indicated by markers

Table S5. Crystal and refinement parameters at room temperature for compositions in the $Li_{4-2x}Ge_{1-x}Mo_xO_4$ system.

x	x = 0.0	x = 0.2	x = 0.5	x = 1.0
Chemical formula	Li_4GeO_4	$Li_{3.6}Ge_{0.8}Mo_{0.2}O_4$	$Li_3Ge_{0.5}Mo_{0.5}O_4$	Li_2MoO_4
M_r (g mol^{-1})	164.37	166.26	169.10	173.82
Crystal system	Orthorhombic	Orthorhombic	Orthorhombic	Rhombohedral
Space group	*Cmcm*	*Pnma*	$Pmn2_1$	*R*-3
Unit cell dimensions (Å)	a = 7.77330(9)<br>b = 7.37177(9)<br>c = 6.05917(7)	a = 10.9419(3)<br>b = 6.2974(2)<br>c = 5.1506(1)	a = 6.4275(3)<br>b = 5.5300(2)<br>c = 4.9192(2)	a = 14.3425(3)<br>c = 9.5946(2)
Volume （Å^3）	347.21(1)	354.90(3)	174.85(2)	1709.25(8)
Z	4	4	2	18
Density (calculated)	3.144 g/cm^3	3.111 g/cm^3	3.212 g/cm^3	3.040 g/cm^3
R-factors (neutron back scattering)	R_{wp} = 0.0166<br>R_p = 0.0245<br>R_{ex} = 0.0043<br>R_F^2 = 0.0528	R_{wp} = 0.0080<br>R_p = 0.0113<br>R_{ex} = 0.0042<br>R_F^2 = 0.0409	R_{wp} = 0.0117<br>R_p = 0.0179<br>R_{ex} = 0.0058<br>R_F^2 = 0.0464	R_{wp} = 0.0248<br>R_p = 0.0347<br>R_{ex} = 0.0059<br>R_F^2 = 0.0264
R-factors (neutron 90°)	R_{wp} = 0.0202<br>R_p = 0.0336<br>R_{ex} = 0.0031<br>R_F^2 = 0.0290	R_{wp} = 0.0129<br>R_p = 0.0176<br>R_{ex} = 0.0027<br>R_F^2 = 0.0870	R_{wp} = 0.0159<br>R_p = 0.0188<br>R_{ex} = 0.0037<br>R_F^2 = 0.1091	R_{wp} = 0.0212<br>R_p = 0.0332<br>R_{ex} = 0.0032<br>R_F^2 = 0.0217
R-factors (X-ray)	R_{wp} = 0.1226<br>R_p = 0.0908<br>R_{ex} = 0.0195<br>R_F^2 = 0.1170	R_{wp} = 0.0890<br>R_p = 0.0697<br>R_{ex} = 0.0462<br>R_F^2 = 0.0940	R_{wp} = 0.0929<br>R_p = 0.0718<br>R_{ex} = 0.0474<br>R_F^2 = 0.0918	R_{wp} = 0.0972<br>R_p = 0.0740<br>R_{ex} = 0.0452<br>R_F^2 = 0.1031
Total R-factors	R_{wp} = 0.0245<br>R_p = 0.0901	R_{wp} = 0.0123<br>R_p = 0.0644	R_{wp} = 0.0160<br>R_p = 0.0664	R_{wp} = 0.0237<br>R_p = 0.0716
χ^2	29.36	8.384	7.485	17.95
No. of variables	116	134	122	135
No. of profile points neutron back scattering	4508	3483	3426	2826
neutron 90°	2252	2058	2078	1762
X-ray	3290	3290	3290	3440

Table S6. Refined structural parameters for Li_4GeO_4.

Atom	Position	*x*	*y*	*z*	U_{iso} (Å^2)	Occ.
Li1	8*e*	0.1620(1)	0.0	0.0	0.0102(2)	1.0
Li2	8*g*	0.3529(1)	0.2264(1)	0.25	0.0129(2)	1.0
Ge	4*c*	0.0	0.33896(4)	0.25	0.00536(6)	1.0
O1	8*f*	0.0	0.20370(4)	0.00905(6)	0.00753(6)	1.0
O2	8*g*	0.19338(5)	0.46255(5)	0.25	0.00782(6)	1.0

Table S7. Refined structural parameters for $Li_{3.6}Ge_{0.8}Mo_{0.2}O_4$

Atom	Site	*x*	*y*	*z*	U_{iso} (Å^2)	Occ.
Li1	4*c*	0.450(2)	0.75	0.151(4)	0.029(2)	0.33(2)
Li1a	4*c*	0.401(1)	0.75	0.212(2)	0.029(2)	0.67(2)
Li2	8*d*	0.1616(4)	-0.0021(6)	0.3316(6)	0.0148(6)	0.812(6)
Li2a	8*d*	0.181(2)	0.058(2)	0.134(3)	0.0148(6)	0.188(6)
Li3	4*c*	0.204(2)	0.25(18)	-0.033(5)	0.086(6)	0.42(1)
Li4	4*c*	0	0	0.5	0.086(6)	0.18(1)
Ge	4*c*	0.4132(1)	0.25	0. 3355(2)	0.0117(2)	0.8
Mo	4*c*	0.4132(1)	0.25	0. 3355(2)	0.0117(2)	0.2
O1	8*d*	0. 3351(1)	0. 0216(1)	0. 2216(2)	0.0158(2)	1.0
O2	4*c*	0. 0855(2)	0.75	0. 1768(3)	0.0154(3)	1.0
O3	4*c*	0. 0643(1)	0.25	0. 2756(3)	0.0150(3)	1.0

Table S8. Refined structural parameters for $Li_3Ge_{0.5}Mo_{0.5}O_4$

Atom	Site	*x*	*y*	*z*	U_{iso} (Å^2)	Occ.
Li1	4*b*	0.2405(6)	0.332(1)	1.003(1)	0.0261(6)	1.0
Li2	2*a*	0.5	0.827(1)	0.968(1)	0.0140(10)	1.0
Ge	2*a*	0.0	0.8282(2)	-0.0014(0)	0.0098(2)	0.5
Mo	2*a*	0.0	0.8282(2)	-0.0014(0)	0.0098(2)	0.5
O1	4*b*	0.2286(2)	0.6772(3)	0.8899(4)	0.0148(2)	1.0
O2	2*a*	0.0	0.1386(3)	0.8940(5)	0.0130(3)	1.0
O3	2*a*	0.5	0.1714(5)	0.8564(5)	0.0200(4)	1.0

Table S9. Refined structural parameters for Li_2MoO_4

Atom	Site	*x*	*y*	*z*	U_{iso} (Å^2)	Occ.
Li1	18*f*	0.1372(3)	0.4526(3)	0.2488(8)	0.0159(5)	1.0
Li2	18*f*	0.3088(2)	0.8546(2)	0.5793(8)	0.0152(5)	1.0
Mo	18*f*	0.11837(5)	0.64731(5)	0.4160(2)	0.0090(2)	1.0
O1	18*f*	0.00488(7)	0.66480(9)	0.4142(3)	0.0164(2)	1.0
O2	18*f*	0.23427(7)	0.77716(7)	0.4162(2)	0.0181(2)	1.0
O3	18*f*	0.11813(9)	0.57852(8)	0.2629(2)	0.0147(2)	1.0
O4	18*f*	0.11852(8)	0.57847(7)	0.5691(2)	0.0175(2)	1.0

Table S10. Significant contact distances (Å) and angles (°) for Li_4GeO_4

Ge-O1 1.7680(4) × 2<br>Ge-O2 1.7577(4) × 2	Li1-O1 1.9607(7) × 2<br>Li1-O2 1.9063(6) × 2	Li2-O1 2.0092(7) × 2<br>Li2-O2 2.1373(12)<br>Li2-O2′ 1.9780(12)
Mean Ge-O 1.763	Mean Li-O 1.983	
O1-Ge-O1 111.34(3)<br>O1-Ge-O2 106.997(8) × 4<br>O2-Ge-O2 117.56(3)	O1-Li1-O1 100.06(5)<br>O1-Li1-O2 117.866(14) × 2<br>O1-Li1-O2′ 106.861(15) × 2<br>O2-Li1-O2 107.75(5)	O1-Li2-O1 102.74(5)<br>O1-Li2-O2 96.97(4) × 2<br>O1-Li2-O2′ 110.83(4) × 2<br>O2-Li2-O2′ 134.06(6)

Table S11. Significant contact distances (Å) and angles (°) for $Li_{3.6}Ge_{0.8}Mo_{0.2}O_4$

Ge/Mo-O1	1.7728(11) × 2	Li1-O1	2.156(15)× 2	Li1a-O1	1.857(5) × 2
Ge/Mo-O2	1.7582(14)	Li1-O2	1.723(18)	Li1a-O2	2.098(11)
Ge/Mo-O3	1.7497(19)	Li1-O3	1.941(19)	Li1a-O3	2.280(12)
Li2-O1	1.986(4)	Li2a-O1	1.765(18)		
Li2-O1	2.0127(35)	Li2a-O1	2.191(15)		
Li2-O2	1.941(4)	Li2a-O2	2.214(13)		
Li2-O3	1.933(4)	Li2a-O3	1.899(17)		
Li3-O1	2.415(20)				
Li3-O1	2.171(16)	Li4-O1	2.1394(11) × 2		
Li3-O1	2.415(20)	Li4-O2	2.4749(11) × 2		
Li3-O1	2.171(16)	Li4-O3	2.0758(10) × 2		
Li3-O2	2.742(23)				
Li3-O3	2.208(24)				
Mean Ge/Mo-O	1.76(1)	Mean Li-O	2.06(7)		
O1-Ge/Mo -O1	108.43(8)	O1-Li1-O1	105.0(10)	O1-Li1a-O1	134.2(7)
O1-Ge/Mo -O2	109.56(6)	O1-Li1-O2	114.6(6)	O1-Li1a-O2	111.51(29)
O1-Ge/Mo -O3	110.32(5)	O1-Li1-O3	96.8(5)	O1-Li1a-O3	95.19(33)
O1-Ge/Mo -O2	109.56(6)	O1-Li1-O2	114.6(6)	O1-Li1a-O2	111.51(29)
O1-Ge/Mo -O3	110.32(5)	O1-Li1-O3	96.8(5)	O1-Li1a-O3	95.19(33)
O2-Ge/Mo -O3	108.64(9)	O2-Li1-O3	125.7(15)	O2-Li1a-O3	96.3(5)
O1-Li2-O1	105.80(17)	O1-Li2a-O1	107.0(8)		
O1-Li2-O2	110.70(18)	O1-Li2a-O2	108.1(8)		
O1-Li2-O3	114.96(20)	O1-Li2a-O3	128.9(8)		
O1-Li2-O2	111.62(16)	O1-Li2a-O2	81.8(5)		
O1-Li2-O3	102.04(17)	O1-Li2a-O3	117.8(9)		
O2-Li2-O3	111.28(20)	O2-Li2a-O3	101.8(7)		

Table S12. Significant contact distances (Å) and angles (°) for $Li_3Ge_{0.5}Mo_{0.5}O_4$

Ge/Mo-O1	1.7729(13)	Li1-O1	1.987(7)	Li2-O1	1.9681(34)
Ge/Mo-O1	1.7729(13)	Li1-O1	1.915(6)	Li2-O1	1.9681(34)
Ge/Mo-O2	1.7920(23)	Li1-O2	1.956(5)	Li2-O2	2.105(6)
Ge/Mo-O	1.7600(26)	Li1-O3	2.024(5)	Li2-O3	1.984(7)
Mean Ge/Mo-O	1.77(1)	Mean Li-O 1.98(9)			
O1-Ge/Mo -O1	111.97(12)	O1-Li1-O1	108.00(30)	O1-Li2-O1	124.8(4)
O1-Ge/Mo -O2	111.39(7)	O1-Li1-O2	114.75(27)	O1-Li2-O2	103.46(17)
O1-Ge/Mo -O3	107.59(8)	O1-Li1-O3	110.76(27)	O1-Li2-O3	110.46(18)
O1-Ge/Mo -O2	111.39(7)	O1-Li1-O2	109.80(31)	O1-Li2-O2	103.46(17)
O1-Ge/Mo -O3	107.59(8)	O1-Li1-O3	104.87(23)	O1-Li2-O3	110.46(18)
O2-Ge/Mo -O3	106.60(13)	O2-Li1-O3	108.21(31)	O2-Li2-O3	100.84(28)

Table S13. Significant contact distances (Å) and angles (°) for Li_2MoO_4

Mo-O1	1.7667(12)	Li1-O1	1.9078(34)	Li2-O1	1.9712(30)
Mo-O2	1.7709(10)	Li1-O2	2.003(7)	Li2-O2	1.907(7)
Mo1-O3	1.7683(20)	Li1-O3	1.9616(30)	Li2-O4	2.0059(34)
Mo-O4	1.7703(22)	Li1-O3	1.982(7)	Li2-O4	1.988(7)
Mean Ge/Mo-O	1.77(2)	Mean Li-O 1.97(9)			
O1-Mo-O2	107.32(5)	O1-Li1-O2	110.48(28)	O1-Li2-O2	114.68(30)
O1-Mo-O3	109.05(13)	O1-Li1-O3	108.07(15)	O1-Li2-O4	99.08(14)
O1-Mo-O4	110.20(13)	O1-Li1-O3	107.33(29)	O1-Li2-O4	104.97(27)
O2-Mo-O3	109.06(10)	O2-Li1-O3	106.30(29)	O2-Li2-O4	108.01(28)
O2-Mo-O4	108.88(10)	O2-Li1-O3	113.04(16)	O2-Li2-O4	118.40(17)
O3-Mo-O4	112.22(4)	O3-Li1-O3	111.55(27)	O4-Li2-O4	109.98(27)
